# Disruption of hnRNP A2-mediated RNA dynamics by amyloid-β drives MBP increase in Alzheimer’s disease

**DOI:** 10.1007/s00018-025-05823-5

**Published:** 2025-08-02

**Authors:** Adhara Gaminde-Blasco, Rodrigo Senovilla-Ganzo, Uxue Balantzategi, Maialen Martinez-Preciado, Estibaliz Capetillo-Zarate, Fernando García-Moreno, Carlos Matute, Jimena Baleriola, Elena Alberdi

**Affiliations:** 1 Department of Neuroscience, University of the Basque Country (UPV/EHU), Leioa, 48940 Spain; 2 Achucarro Basque Center for Neuroscience, Leioa, 48940 Spain; 3 Ikerbasque, Basque Foundation for Science, Bilbao, 48009 Spain; 4 CIBERNED, Leioa, 48940 Spain

**Keywords:** Oligodendrocytes, MBP, HnRNP A2, Calcium homeostasis, Amyloid-β peptide, Alzheimer´s disease

## Abstract

Oligodendrocyte dysfunction, myelin degeneration, and white matter changes are critical events in the cognitive decline of Alzheimer’s disease (AD). Amyloid-β peptide (Aβ), a hallmark of AD, disrupts oligodendrocyte and myelin homeostasis, through mechanisms that remain poorly understood. Here, transcriptomic profiling of Aβ-exposed oligodendrocytes revealed widespread gene expression changes, particularly in RNA-related processes. Among these, hnRNP A2, a key regulator of RNA transport and myelin protein regulation, was aberrantly upregulated in hippocampal oligodendrocytess from AD patients with high Aβ levels, from AD mouse models, and in Aβ-treated oligodendrocytes. RNA-immunoprecipitation sequencing of the hnRNP A2 interactome revealed Aβ-induced changes in mRNA interactions, particularly enriched binding to *Mbp* and *Mobp*, indicating impaired RNA metabolism of myelin components. Furthermore, Aβ, through hnRNP A2 disruption, increased the number, cargo and dynamics of *Mbp*- and *Mobp*-containing granules, enhanced MBP and MOBP synthesis, and decreased oligodendroglial voltage-gated Ca^2+^ influx in an MBP-dependent manner. These findings suggest that Aβ-induced dysregulation of hnRNP A2 impairs RNA metabolism and myelin protein synthesis, altering the intracellular Ca^2+^ homeostasis critical for oligodendrocyte function.

**Graphical abstract:**

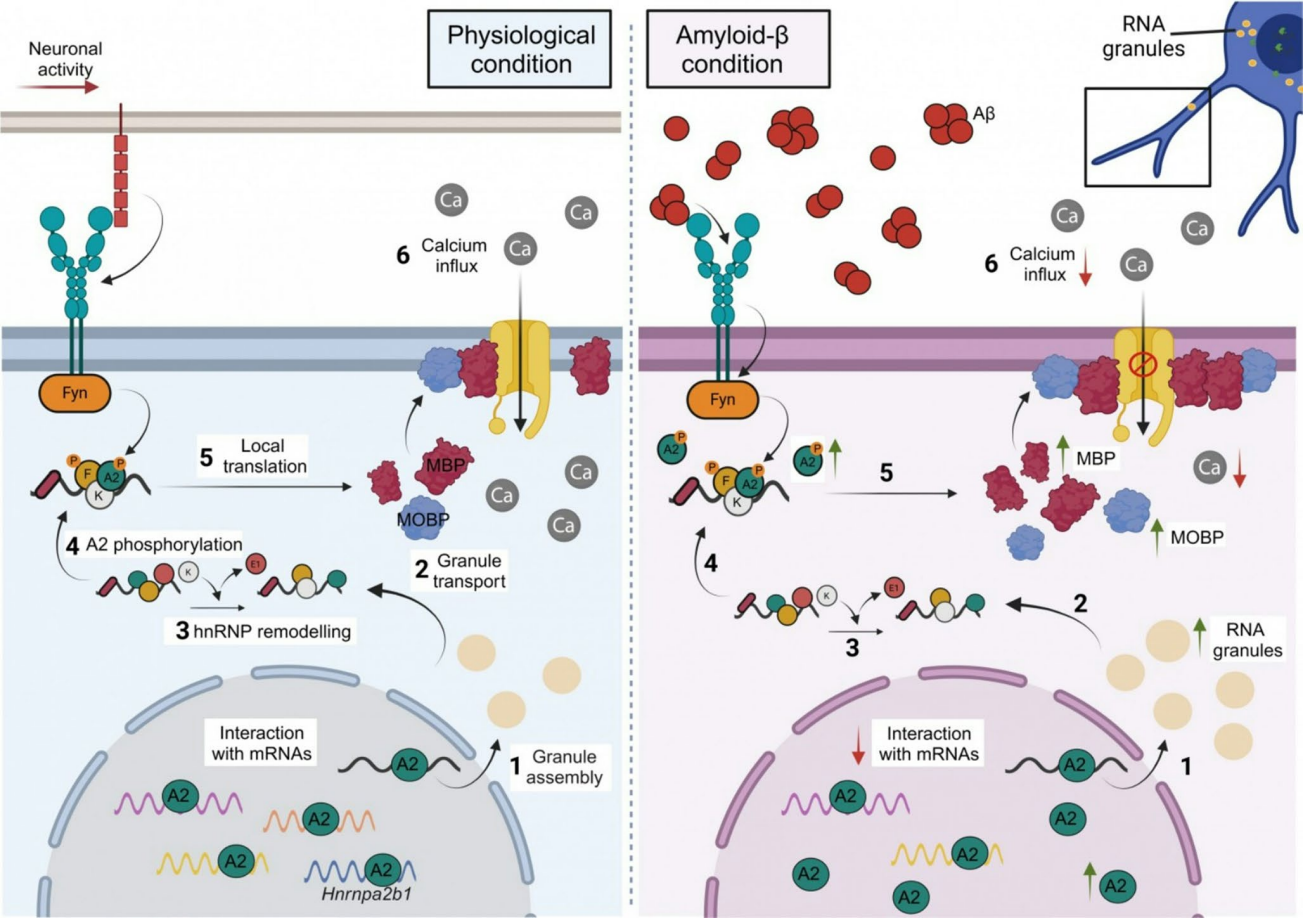

**Supplementary Information:**

The online version contains supplementary material available at 10.1007/s00018-025-05823-5.

## Introduction

The human brain consists of more than 50% of white matter (WM), which is composed of nerve fibres with varying degrees of myelination that allow transfer of signals across various areas of the central nervous system (CNS). Oligodendrocytes (OLs) are one of the major cell types in the WM. They play a key role by extending membrane processes to wrap around the axons of multiple neurons, creating and maintaining a specialised multilamellar lipid structure known as myelin. Myelin not only prevents ion leakage but also enhances nerve conduction efficiency and speed. In addition to insulation, OLs provide trophic and metabolic support to axons [1].

The protein content of myelin is dominated by proteolipid protein (PLP) and myelin basic protein (MBP). The formation and maintenance of myelin therefore require the targeted delivery of large amounts of PLP and MBP to the axon-glia contact site, a process precisely regulated in time and space. *Mbp* mRNA is transported from the nucleus within RNA granules to the distal regions of oligodendrocytic processes and to the myelin membrane, where it is locally translated, preventing ectopic expression within the cell [2]. In OLs, this process is governed by the heterogeneous nuclear ribonucleoprotein A2/B1 (hnRNP A2) with the help of other hnRNPs [3]. Beyond its role in *Mbp* transport and translation, hnRNP A2 is one of the most abundant RNA binding proteins (RBPs) and a key regulator of RNA metabolism, controlling multiple transcripts simultaneously by recognising specific motifs [4].

The diverse roles of the nucleo-cytoplasmic RBPs suggest that they are critical coordinators of mRNA metabolism. Recently, a new focus is placed on the role of RNA processing and its impact on neurodegenerative diseases like Alzheimer´s disease (AD) [5]. One of the hallmarks of AD is the presence of extracellular aggregates of amyloid-β peptide (Aβ), and Aβ oligomer-induced changes in OLs and myelin [6]. Furthermore, myelin and oligodendrocyte abnormalities are associated with increased Aβ peptide levels in AD mouse models [7] and in the brains of AD patients [8].

In the last few years, OLs have been identified as a source of Aβ in vivo and thus, active contributors to the disease [9]. Moreover, in AD, OL alterations precede neuronal impairment, and the loss of OLs may lead to cognitive deficits [10]. Recently, multi-omic studies in Alzheimer’s patients found that MYRF -a key transcription factor involved in regulating myelination- was upregulated in the early stages of the disease but decreased in later stages [11]. This suggests that alterations in OL activity may play an important role in the initial phases of AD. However, the mechanisms by which OLs become dysfunctional and lead to AD pathology remain unknown. In vitro studies have shown that Aβ becomes toxic when it oligomerises [12] and can directly alter myelination [13]. Aβ changes MBP expression levels via integrin β1 and Fyn kinase signalling [13], but the functional consequences of MBP overexpression in OLs are not yet understood.

This study highlights significant transcriptomic changes in Aβ-exposed OLs, with a focus on RNA metabolism pathways. HnRNP A2 showed aberrant upregulation in OLs from AD brains high Aβ burden, AD mouse models, and Aβ-treated OLs. RNA immunoprecipitation sequencing (RIP-seq) revealed altered hnRNP A2 interactions, with diminished binding to some mRNAs but enriched binding to *Mbp* and *Mobp*, suggesting disrupted RNA metabolism that might contribute to myelination alteration. Furthermore, Aβ exposure, through hnRNP A2 modifications, increased the dynamics and number of *Mbp-* and *Mobp*-containing granules, enhancing local synthesis of MBP and MOBP and reducing oligodendroglial voltage-gated Ca²⁺ influx in a MBP-dependent manner. These findings implicate hnRNP A2 in AD-related myelin dysfunction.

## Materials and methods

### Animals

Experiments were performed in Sprague Dawley rats, C57BL6/J mice, and in the triple transgenic mouse model of Alzheimer´s disease (3xTg-AD) and wild type mice as controls (B6129SF2/J) [14]. All experimental procedures were conducted under the supervision and approved by the Ethic Committees of the University of the Basque Country (UPV/EHU). All possible efforts were made to minimize animal suffering and the number of animals used.

### Oligodendrocyte primary culture, transfections and infections

Highly enriched OPCs were prepared from mixed glial cultures obtained from newborn (P0–P2) Sprague–Dawley rat forebrain cortices as previously described [15]. Briefly, forebrains were removed from the skulls and the cortices were enzymatically and mechanically digested. Then, tissue was plated in Iscove’s Modified Dulbecco’s Medium supplemented with 10% fetal bovine serum. The mixed glial cells were grown in poly-D-lysine (PDL) treated T75 flasks until they were confluent (8–10 days). Microglia were separated from the cultures by shaking the flasks on a rotary shaker. OPCs were isolated by additional shaking for 18 h (200 rpm, 37 °C, 5% CO_2_). OPCs were seeded on to PDL-coated coverslips and were maintained at 37 °C and 5% CO_2_ for 3 days in a chemically defined maturation SATO medium. At 3–4 days in vitro 88% of the cells are mainly OLs that express MBP [15].

In transfection assays, primary oligodendroglial cells were nucleofected with 20 µM siRNA pools (non-targeting siRNA, Dharmacon #D-001810-10-05; *Mbp*-targeting siRNA, Dharmacon #L-089810-02-0005, *Hnrnpa2b1*-targeting siRNA, Dharmacon #L-094949-02-0005) using AmaxaTM Basic NucleofectorTM kit (#VPI-1006, Lonza) for Primary Mammalian Glial cells following the manufacturer´s instructions.

For viral infection, two adeno-associated viruses (AAV) were used, both carrying MBP promoter and bound to the green fluorescent protein (GFP), as a reporter, but one of them with the MBP gene (AVV8-pMBP-MBP-IRES-GFP) and the other one empty (AAV8-pMBP_GFP). After one day in vitro, OLs were infected with either virus at a MOI of 120,000 viral genomes (vg) per cell.

### Preparation of amyloid-β peptide

Oligomeric amyloid-β (Aβ1–42) was prepared as reported previously [13]. Briefly, Aβ1–42 (Bachem, Germany) was initially dissolved in hexafluoroisopropanol (Sigma) to a concentration of 1 mM. For the aggregation protocol, the peptide was resuspended in dry dimethylsulfoxide (DMSO; Sigma) (Aβ1–42 5 mM). Hams F-12 (PromoCell) was added to adjust the final peptide concentration to 100 µM to obtain oligomers (4 °C for 24 h). As controls, cells were treated with the vehicle (DMSO + Hams F-12). The characterization of this Aβ preparation by transmission electron microscopy showed mainly Aβ oligomers and very few protofibrils [16].

### Paraffin-embedded human sections

Paraffin-embedded human sections (obtained from Biobanc, Hospital Clinic, IDIBAPS, Barcelona, Spain) were deparaffinised and rehydrated by immersing in xylene followed by incubations with alcohol solutions (100%, 96% and 75% diluted in H_2_O) in TBS for 10 min each. Samples were then boiled in antigen retriever R-Universal Buffer (Aptum) for 20 min. After retrieval, samples were washed in TBS 3 times and blocked in 4% BSA in TBS for 1 h at room temperature (RT) and incubated overnight (O/N) with the following primary antibodies in blocking solution, hnRNP A2 (sc-374053, 1:1000, Santa Cruz) and Olig2 (MABN50, 1:500, Millipore). Then, samples were washed in TBS twice and incubated with Alexa-conjugated secondary antibodies (1:1000) and DAPI (4 µg/ml, Sigma-Aldrich) for 1 h at RT. Samples were washed in TBS and treated with Autofluorescence Eliminator Reagent according to the manufacturer’s instructions (#2160, Millipore) to reduce lipofuscin-like autofluorescence. Finally, sections were washed and mounted with Fluoromont-G mounting medium (SouthermBiotech). Single-stack images were acquired using a a Slide Scanner Panoramic MIDI II (3D Histech) with a 20X objective. Image analysis was performed using CaseViewer (CV) and ImageJ/Fiji software. The CV program was used to select three specific regions of interest (ROIs) within the hippocampus, with all ROIs having the same perimeter and area for each patient. To analyse the mean intensity of the hnRNP A2 signal, ImageJ/Fiji was used with standardized parameters to ensure consistent cell selection. These parameters included a radius of 0.6 pixels, a cell size range from 15 μm to infinity, and a circularity between 0.40 and 1.00. After applying these criteria, oligodendrocyte-positive cells were identified. The corresponding image of the same sample and region stained with the hnRNP A2 marker was then examined to match the positive oligodendrocytes with their hnRNP A2 mean intensity. Representative images were acquired using a Leica SP8 confocal microscope (Leica) with a 40x oil-immersion objective.

### Western blot

OLs were exposed to Aβ peptide as indicated. Cells were scraped in sodium dodecyl sulfate (SDS) sample buffer on ice to enhance the lysis process and avoid protein degradation. Samples were boiled at 95 °C for 8 min, size-separated in 4–20% polyacrylamide-SDS Criterion TGX Precast gels (Bio-Rad) and transferred to Trans-Blot Turbo Midi Nitrocellulose Transfer Packs (Bio-Rad). Membranes were blocked in 5% BSA in Tris-buffered saline/0.05% Tween‐20 and proteins were detected by specific primary antibodies against MBP (#SMI 99, 1:1000; Biolegend), MOBP (bs-11184R, 1:1000, Bioss), hnRNP A2/B1 (sc-374053, 1:1000, Santa Cruz), hnRNP F (sc-32310, 1:1000, Santa Cruz), hnRNP K (RN019P, 1:1000, MBL), hnRNP E1 (RN024P, 1:1000, MBL), GAPDH (#2118S, 1:5000, Cell Signalling) and β‐actin (#A20066, 1:5000; Sigma‐Aldrich). Membranes were incubated with horseradish peroxidase-conjugated secondary antibodies (1:5000) or fluorescence secondary antibodies (1:5000). The protein bands were detected with a ChemiDoc XRS Imaging System (Bio-Rad), and quantified by using Image Lab 6.0.1 (Bio-Rad) software. Protein preparation from 3xTg-AD mice and human samples was prepared as previously described [13].

### Immunochemistry

Cells were fixed in 4% paraformaldehyde, 4% sucrose for 15 min, washed with PBS and blocked in 4% normal goat serum, 0.1% Triton X-100 in PBS (blocking buffer) for 1 h and incubated O/N at 4 °C with primary antibodies against MBP (#SMI 99, 1:1000; Biolegend), hnRNP A2/B1 (sc-374053, 1:500, Santa Cruz), hnRNP F (sc-32310, 1:500, Santa Cruz), hnRNP K (RN019P, 1:500, MBL) and hnRNP E1 (RN024P, 1:500, MBL). Samples were washed in PBS and incubated with fluorochrome-conjugated secondary antibodies (1:500) in blocking buffer for 1 h at RT. Cell nuclei were detected by incubation with DAPI (4 µg/ml, Sigma‐Aldrich) and mounted with Fluoromount‐G (SouthermBiotech).

40 μm-thick coronal sections were washed in 0.1 M PB and were then boiled in antigen retriever R-Universal Buffer (Aptum) for 20 min. The sections were then washed three times in 0.1 M PBS and treated with ice-cold 100% EtOH for 10 min. After retrieval, samples were washed in 0.1 M PBS 3 times and blocked in 10% NGS, 0.1% Triton X-100 in PBS for 30 min at RT and incubated O/N with the following primary antibodies in blocking solution, hnRNP A2 (sc-374053, 1:1000, Santa Cruz) and Olig2 (MABN50, 1:500, Millipore). Then, samples were washed in PBS twice and incubated with fluorochrome-conjugated secondary antibodies and DAPI (4 µg/ml, Sigma-Aldrich) for 1 h at RT. Samples were washed in PBS and mounted with Fluoromont-G mounting medium (SouthermBiotech). Images were acquired on a Leica Stellaris 5 (DM6 B CS) confocal microscope (Leica) with a 40X oil-immersion objective.

### Intrahippocampal injection in adult mice

10-week-old male mice (C57BL6/J) were subjected to intrahippocampal injections in the right dentate gyrus (DG). For the surgery, animals were anesthetised with 0.3 ml of Avertin, with addition of 0.1 ml if needed. Mice were immobilized with a stereotaxis apparatus and injected with the corresponding preparations: vehicle or 3 µL of Aβ at 10 µM. Brains were extracted and postfixed with the same fixative solution for 4 h at RT, placed in 30% sucrose in 0.1 M PBS pH 7.5 at 4 °C, and then stored in cryoprotectant solution (30% ethylene glycol, 30% glycerol and 10% 0.4 M PB in dH_2_O) at −20 °C.

### Human oligodendroglioma cell line culture

The HOG cell line, was kindly provided by Dr. U. Gómez-Pinedo (Hospital Clínico San Carlos, Madrid, Spain). HOG cells were grown at 37 °C and 5% CO2 and cultured in DMEM (ThermoFisher) with 4.5 g/L of glucose, supplemented with 1 mM sodium pyruvate (ThermoFisher), 10% heat-inactivated FBS (ThermoFisher), and 1% pen/strep antibiotics (ThermoFisher). To induce their differentiation, cells were seeded in their growth medium (day 0). After 24 h (day 1) the growth medium was replaced by the differentiation medium (DM) [17]. Cells were cultured until day 5 to obtain differentiated HOG (dHOG), during which DM was replaced at day 3.

### Immunoprecipitation

1 µg of agarose conjugated mouse anti-pTyr (PY99, sc-7020 AC, Santa Cruz) and mouse anti-IgG (sc-2342 AC, Santa Cruz) were used. Cells were washed with cold PBS and scraped with RIPA lysis buffer (ThermoFisher Scientific) supplemented with protease and phosphatase inhibitor cocktail (ThermoFisher Scientific). Samples were placed on ice for 10 min and centrifuged 5 min at 12,000 x g. The supernatant was added (1/10 was saved for input) to the agarose conjugated with anti-pTyr or anti-IgG and incubated for 2 h at 4 °C to avoid unspecific interactions. The lysate-antibody‐beads complex was centrifuged at 2,000 x g for 2 min and washed three times with RIPA lysis buffer and once by PBS followed by centrifugation to obtain the immunocomplex. Finally, protein elution was carried out in 2x sample buffer after boiling the samples at 95 °C for 5 min and centrifuged at 12,000 x g for 1 min. Proteins were analysed by Western blot.

### RNA-immunoprecipitation (RIP)

RIP was performed as previously described with some modifications [18]. In brief, cells were scrapped in polysome lysis buffer (100 mM KCl, 5 mM MgCl2, 10 mM HEPES pH 7.0, 0.5% NP-40, 1 mM DTT, 100 units/ml RNase OUT, 1X Protease Inhibitor Cocktail) and incubated with a suspension of 1 µg agarose conjugated mouse anti-hnRNPA2 antibody (sc-374053 AC, Santa Cruz) and mouse anti-IgG (sc-2342 AC, Santa Cruz). RNA was isolated following a TRIzol RNA isolation protocol (ThermoFisher Scientific). An equal volume of extracted RNA from each sample was then used for cDNA synthesis and analysed by quantitative PCR. Data were normalised to a normalisation factor obtained in geNorm Software through the analysis of the expression of two housekeeping genes. *Mbp*, *Mobp* and *Tau* mRNA enrichment was examined by relativizing the RIP fraction values to the normalised input values.

For, the hnRNP A2 interactome analysis, total RNA from three biological replicates of hnRNP A2 and IgG IPs, as well as three replicates of input mRNA were evaluated using Agilent RNA 6000 Pico Chips (Agilent Technologies, Cat. #5067 − 1513). Sequencing libraries were prepared using “SMARTer Stranded Total RNA-seq Kit v2– Pico Input Mammalian” kit (Takara Bio USA, Cat. # 634411), following “SMARTer Stranded Total RNA-seq Kit v2– Pico Input Mammalian User Manual (Rev. 050619)”.

### RNA in situ hybridization combined with immunofluorescence

FISH was performed using the RNAScope^®^ Multiplex Fluorescent V2 Assay Kit (Advanced Cell Diagnostics). Cells were fixed in 4% PFA in PBS for 10 min and washed with PBS. FISH was performed according to the manufacturer’s instructions for cultured adherent cells. The rat *Mbp* and *Mobp* transcript probes were designed and synthesized by the manufacturer, and used at 1:50 dilution, except for *Mbp*, which was used undiluted. The transcripts were fluorescently labeled by TSA-based Opal fluorophores Opal570 (1:1500) and Opal650 (1:1500) using the Opal 7 Kit (PerkinElmer). After FISH, immunofluorescence was performed as described above incubating with primary antibody hnRNP A2/B1 (sc-374053, 1:500, Santa Cruz) 2 h at RT. Then, samples were washed in PBS twice and incubated with fluorochrome-conjugated secondary antibodies for 1 h at RT. Finally, samples were washed in PBS and mounted with ProLong™ Gold antifade reagent with DAPI (Invitrogen).

Images were acquired on a Leica Stellaris 5 (DM6 B CS) confocal microscope (Leica) with a 63X oil-immersion objective with LIGHTNING super resolution module. 3–4 z-stack tiles (z step = 0.24 μm) of 10 cells were acquired from each condition. For the colocalization analysis, two approaches were employed. First, the percentage of area showing colocalization between the probe (*Mbp* or *Mobp*) and hnRNP A2 was determined using ImageJ/Fiji with the Autosegment_Coloc.ijm plugin (https://github.com/SoriaFN). In short, maximum intensity projections were generated from z-stack images. Then, images were binarised using consistient threshold values across all samples. Finally, the percentage of *Mbp*- or *Mobp*-positive area colocalizing with hnRNP A2 was calculated.This analysis was performed excluding the nucleus. Second, a 3D colocalization analysis was performed using the Huygens Professional software (24.10 version). The Huygens Colocalization Analyzer module was used to calculate the Pearson colocalization coefficient, providing a quantitative assessment of spatial overlap between the markers in three dimensions. For both analyses, the same thresholding parameters were applied across all conditions to ensure consistency and comparability.

### Quantitative RT-PCR (RT-qPCR)

Total RNA was extracted from cultured OLs using RNA Mini Kit (Quiagen) or TRIzol (ThermoFisher Scientific) according to manufacturer´s instructions. Strand complementary DNA synthesis was carried out with reverse transcriptase Superscript TMIII (Invitrogen) using random primers. Specific primers for *Mbp*, *Hnrnpa2b1*, *Hnrnpf*, *Hnrnpe1* and *Hnrnpk* were obtained from Qiagen. It was newly designed for *Mobp-81a* (5´-CGCTCTCCAAGAACCAGAAG-3´ and 5´-GCTTGGAGTTGAGGAAGGTG-3´). RT-qPCR reactions were carried out with 25 ng of reverse transcribed RNA and 300 nM of primers diluted in Sybr Green Master Mix reagent (Bio-Rad). PCR product specificity was checked by melting curves. Data were normalised to a normalisation factor obtained in geNorm Software through the analysis of the expression of three housekeeping genes.

### RNA-seq

RNA quantity and quality was assessed using Qubit RNA assay kit (Invitrogen) and Agilent 2100 Bioanalyzer (Agilent RNA 6000 Pico Chips). Sequencing libraries were prepared using “SMARTer Stranded Total RNA-seq Kit v2– Pico Input Mammalian” kit (Takara Bio USA, Cat. #634411). The protocol was started with 4–10 ng of total RNA. Paired-end readings were generated from all libraries using HiSeq200 sequencer following Illumina protocols. The base calls or BCL were converted into FASTQ files by utilizing Illumina Inc.’s package bcl2fastq and quality control analysis was performed. Alignment was carried out with STAR v2.7.1 [19] against Ensembl genome of Rattus norvegicus (BN7.2.toplevel ang gtf v105) and expression counts were obtained with htseq-count (-sreverse) [20]. The count matrix was imported to R v4.2.2, where expression levels were normalised and further analysed with DESeq2 [21]. Those genes with less than 5 for the effect of the Aβ-treatment analysis and less than 2 counts for the interactome analysis in more than one sample per group were discarded. The impact of Aβ in OLs whole transcriptome was explored with the following design: ~ SV1 + SV2 + Pair + Experiment + Condition + Experiment: Condition. SV1 and SV2 factors have computed based on Surrogate Variable Analysis [22] to extract variability due to unknown noisy factors such as Aβ oligomerization and others. To explore the effect of Aβ in the whole-cell transcriptome, the following contrast was used: Condition_Aβ_vs_Control. Those genes with p-adjusted value < 0.05 were considered differentially expressed genes.

To obtain the hnRNP A2 interactome, we split our control and Aβ-treated samples in the analysis, but following a shared design model ~ Pair + Experiment. Similarly to other interactome literature [23], contrast list (Experiment_RIP_vs_Input; Experiment_IgG_vs_Input) was carried out. Those genes with p-adjusted value < 0.05 and log2FC greater than0 were identified as significantly interacting with hnRNP A2. For data visualization and functional enrichment: ggplot2 [24], clusterProfiler [25, 26] and ggVennDiagram [27] were used. The code used can be found in https://github.com/rodrisenovilla/Gaminde-Blasco2024.

### Puromycylation-proximity ligation assay (Puro-PLA)

To detect newly synthesised proteins, cells were exposed to puromycin (2 µM, Sigma-Aldrich) for 10 min in the absence or presence of the protein synthesis inhibitor anisomycin (40 µM) for 25 min. After incubation, cells were washed with cold PBS with digitonin and fixed in 4% PFA, 4% sucrose in PBS for 15 min. PLA was conducted following Duolink^®^ PLA Protocol (Sigma-Aldrich). Briefly, as soon as cells were permeabilized and blocked, these were incubated at 4 °C O/N with rabbit anti-MBP (A1664, 1:500, ABclonal), rabbit anti-MOBP (bs-11184R, 1:500, Bioss) and mouse anti-puromycin (MABE343, 1:500, Millipore). Detection of newly synthesised proteins was performed according to the manufacturer’s recommendations (#DUO92101, Sigma-Aldrich). Finally, cells were washed and treated with Alexa FluorTM 488-conjugated phalloidin (A12379, ThermoFisher) in 1% BSA. Images were taken with a Zeiss Apotome 2 (Zeiss) epifluorescence microscope using 63X oil-immersion objective. Image analysis was performed using ImageJ/Fiji software. Phalloidin signal was processed with a Gaussian Blur plugin to create a mask. Employing a “Concentric Circles” plugin, concentric circles at 10 μm intervals emerging from the centre of the cell body were generated and mean values of fluorescence intensity of PLA probes were obtained.

### Calcium imaging

OLs were loaded with 1 µM Fluo-4 AM (Invitrogen) or x-Rhod-1 AM (Invitrogen) in culture medium for 30 min at 37 °C and then washed. OLs were exposed to 25 mM KCl and images were acquired through a 40X oil objective with an inverted Zeiss LSM800 confocal microscope (Zeiss) at an acquisition rate of 1 frame/15 s for 5 min. For data analysis, a homogeneous population of > 10 cells was selected in the field of view and cell somata were selected as ROIs. Background values were always subtracted and data are expressed as F/F0 ± S.E.M (%) in which F represents the fluorescence value for a given time point and F0 represents the mean of the basal fluorescence level.

### Statistical analysis

All data were expressed as mean ± S.E.M. Statistical analyses were performed using absolute values. GraphPad Prism 8.2.1 software was used applying one-way or two-way analysis of variance (ANOVA) followed by Dunnett´s, Sidak´s and Tukey´s post hoc tests for multiple comparisons and two-tailed, unpaired or paired Student’s t test for comparison of two experimental groups. Results from independent patients, animals or independent cultures were treated as biological replicates (*n* ≥ 3). Statistical analysis of Aβ treatment and interactome were conveniently explained in the RNA-seq section.

## Results

### Transcriptomic profiling of Aβ-treated OLs revealed alterations in RNA localisation and ribonucleoprotein complex

**Fig. 1:**
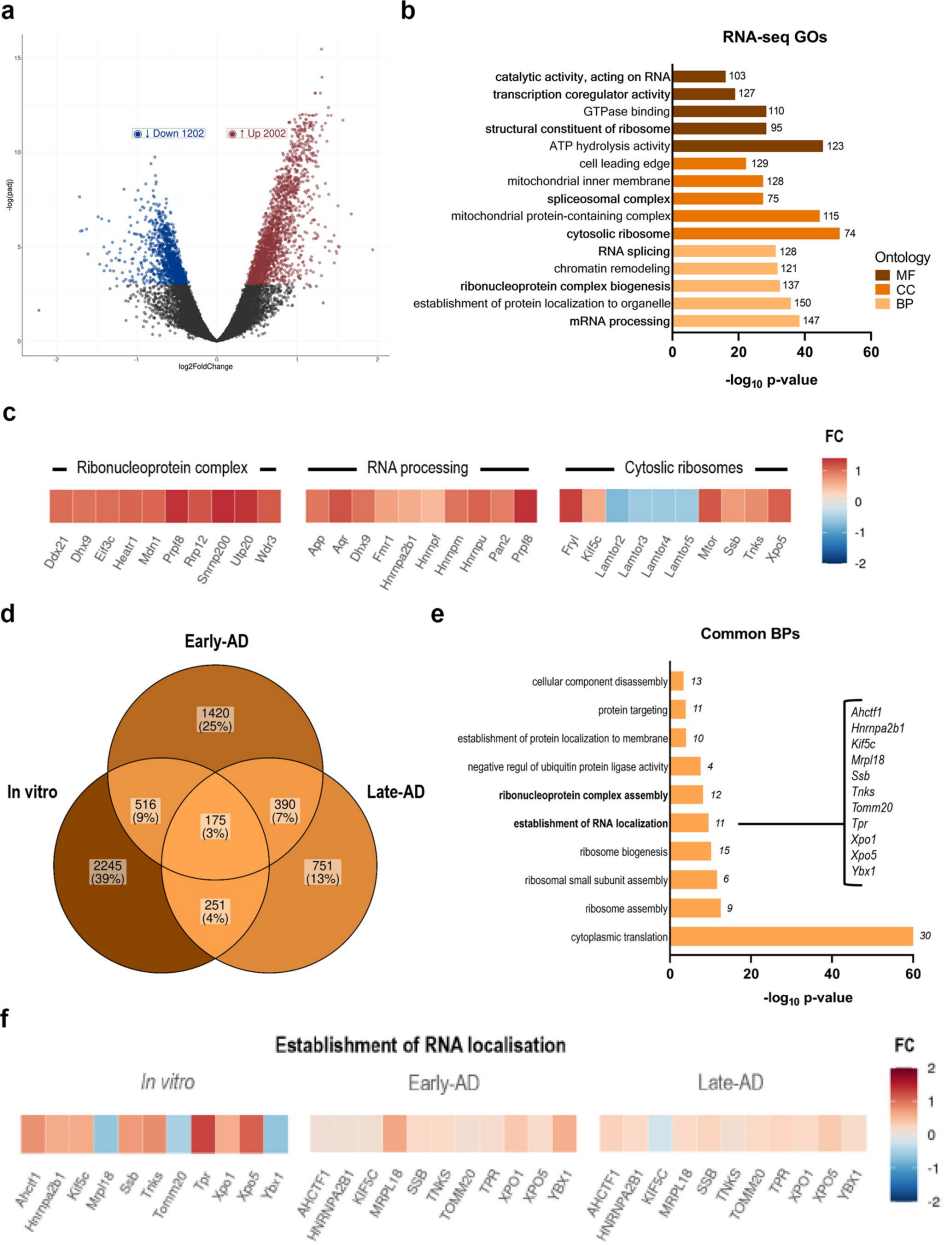
Aβ-treated cells show alterations in RNA metabolism and translation. **a** Volcano plot of differentially expressed genes (DEGs). Analysed genes are scattered by their expression levels (axis X, log10 mean of normalised counts) and fold change (FC) (axis Y, log2FC). Differentially expressed genes (Padj < 0, 05) are coloured based on their FC (blue < 0; red > 0). **b** Gene ontology enrichment analysis of biological process (BP), cellular component (CC) and molecular function (MF) of DEGs and number of genes associated in each GO term. **c** Selected GO terms related to ribonucleoproteins, RNA processing and myelination. Differentially expressed genes (Padj < 0. 05) are coloured based on their FC (red < 0; blue > 0). **d** Venn diagram depicting the overlap of DEGs in vitro vs. human AD patients [28]. Percentage and numbers indicate the genes shared among the datasets. **e** Gene ontology enrichment analysis of the biological processes shared in Aβ and AD condition. **f** Selected GO terms related to the establishment of RNA localisation are shown. DEGs (Padj < 0. 05) are coloured based on their FC (red < 0; blue > 0)

To test the impact of Aβ on the oligodendrocyte transcriptome, we performed bulk RNA-sequencing (RNA-seq) on both control and Aβ-treated (1 µM, 24 h, *n* = 3) primary cultured OLs. We identified 3,204 differentially expressed genes (DEGs) using DeSeq2 (Padj < 0.05; Fig. 1a). Among these, 2,002 genes were upregulated, while 1,202 were downregulated (Fig. 1a, Supplmentary Table S1). To broadly characterize the potential transcriptomic effects of Aβ on molecular processes, we performed Gene Ontology (GO) term enrichment analyses. Notably, we observed a pronounced enrichment of genes associated with RNA processing, ribonucleoprotein complex biogenesis or cytosolic ribosomes (Fig. 1b and Supplementary Fig. S1a, b), suggesting a significant impact of Aβ on mechanisms related to RNA metabolism and translation (Fig. 1c).

Next, we asked whether the gene signatures associated with Aβ identified in vitro, were altered in OLs derived from AD patients. We curated published datasets to obtain gene signatures from oligodendrocyte-lineage cells derived from early- and late-stage human AD patients. These patients were classified based on the present of elevated Aβ levels and other AD-related pathological hallmarks, as well as control individuals with minimal or no Aβ burden or AD pathology [28]. We then analysed the overlap between DEGs obtained from the mentioned datasets and those observed in our in vitro model. We identified 175 DEGs common to the three conditions (Fig. 1d). Importantly, most of them were primarily linked to cytoplasmic translation, ribosome formation, ribonucleoprotein complex assembly or RNA localisation (Fig. 1d and Supplementary Fig. S1c, d), confirming the pathophysiological relevance of RNA metabolism and translation in AD.

Many genetic neurological disorders, including AD, are characterised by mutations in RBPs [29], and defects in RNA metabolism (including splicing, localisation or degradation) and translation are common to all of them [5]. Such defects have been mainly studied in neurons but given the prominence of DEGs related to the aforementioned mechanisms in OLs, we explored the effect of Aβ on RBP levels in cultured OLs. Thus, we focused on the 11 RNAs involved in RNA metabolism shared across all three conditions (Fig. 1f). Specifically we focus on genes that were upregulated in oligodendrocytes treated with Aβ and that showed increased expression correlating with disease progression in AD patient-derived datasets. Among these, candidates included *Xpo5*,* Tpr*,* Ssb*,* Hnrnpa2b1 and Achtf1*, all implicated in general RNA processing functions such as nuclear export (*Xpo5*), nuclear pore complex activity (*Tpr*), RNA binding (*Ssb*), and transcription regulation (*Achtf1*). While some of them, such as *Xpo5* [30] or *Tpr* [31] have been associated with AD, they are not known to be involved in oligodendrocyte-specific functions. In contrast, *Hnrnpa2b1* is one of the most abundant and essential RNA-binding proteins in the CNS, which is known to regulate splicing events in AD [32]. Moreover, it regulates the RNA transport and local translation of myelin-related mRNAs, including MBP and MOBP, which are fundamental for OL function [3]. Previous study has shown that Aβ disrupts MBP expression [13], supporting a mechanism whereby Aβ-induced dysregulation of hnRNP A2 could contribute to OL dysfunction through impaired myelin gene regulation. Interestingly, our analysis revealed that hnRNP A2 is overexpressed in AD and shows a slight progressive increase with disease severity, correlating with higher Aβ accumulation. Therefore, given hnRNP A2’s key role in regulating the RNA transport and translation of two critical myelin proteins, it is likely that alterations in nuclear and/or cytoplasmic hnRNP A2 regulation in OLs contribute to the pathology observed in AD. Based on the literature and our own results, we further focused on studying the regulation of hnRNP A2 in OLs in the context of AD.

### hnRNP A2 levels are increased in hippocampal OLs from AD patients

**Fig. 2:**
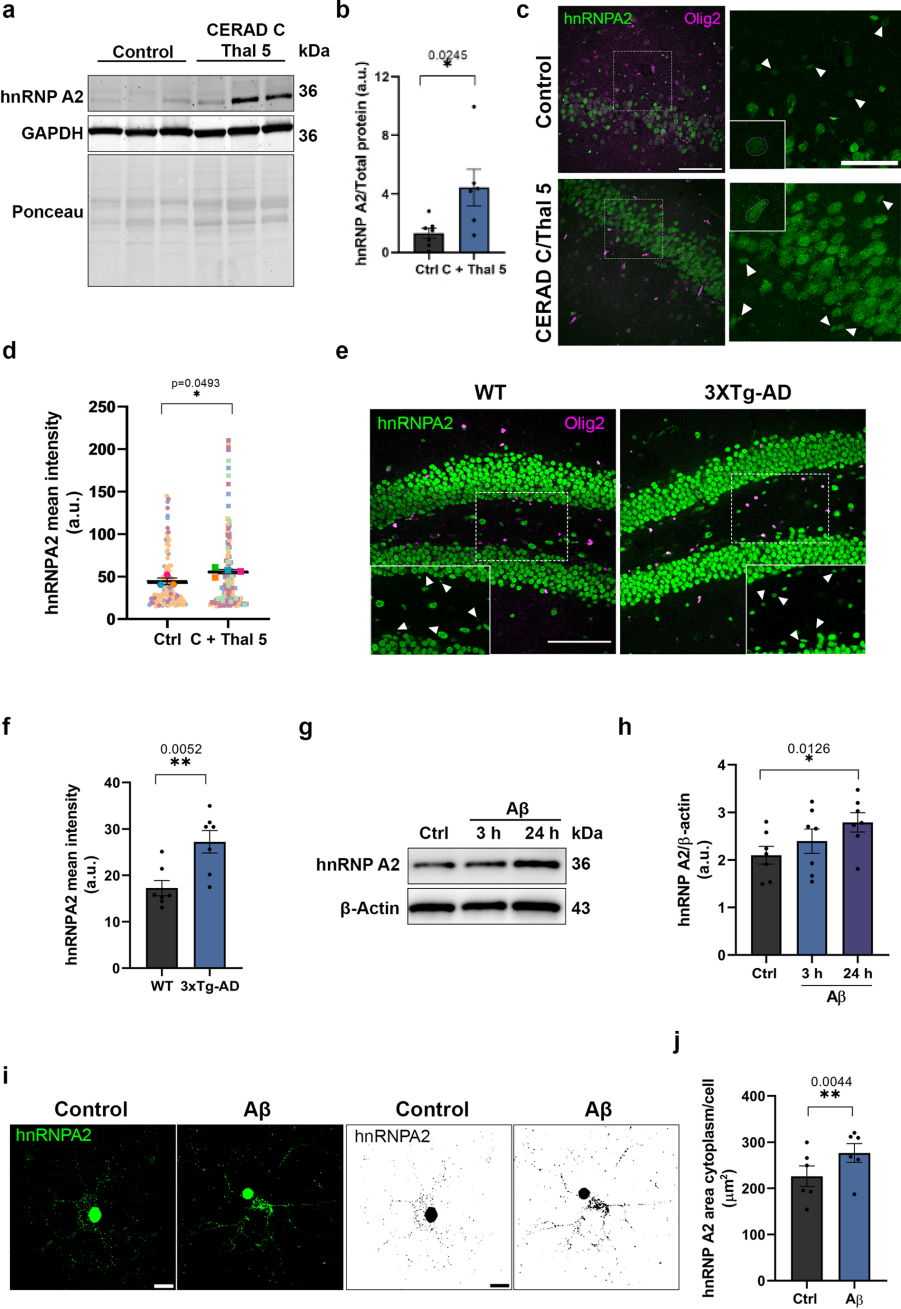
hnRNP A2 expression and localisation changes in oligodendroglial cells in AD brains, AD mouse models and primary oligodendrocyte culture treated with Aβ. **a**, **b** hnRNP A2 expression and relative quantification in human hippocampal lysates from control (*n* = 7) and AD patients (*n* = 6) (CERAD C and Thal 5) normalized to total protein content (Ponceau). **c**, **d** Representative confocal images show the Olig2 (magenta) and hnRNP A2 (green) expression in the hippocampus from controls (*n* = 3) and AD patients (CERAD C and Thal 5) (*n* = 4). Quantification of hnRNP A2 mean intensity signal in Olig2^+^ cells in the human hippocampus. Scale bars, 100 μm and 50 μm. **e**, **f** Representative confocal images of Olig2 (magenta) and hnRNP A2 (green) in the dentate gyrus of 6-month-old of WT (*n* = 7) and 3xTg-AD (*n* = 7) mice. Histogram depicts changes in the mean intensity of hnRNP A2 in oligodendroglia lineage. Scale bar, 100 μm. **g**, **h** HnRNP A2 expression and relative quantification in oligodendrocyte cell extracts normalised to β-actin (*n* = 7). **i**, **j** Representative confocal and binary micrographs of hnRNP A2 in control and Aβ-treated OLs. Histogram depicts changes in the area occupied by hnRNP A2 (*n* = 6). Data indicate means ± S.E.M and dots represent patients (**b**), individual cells and media from the same patient (**d**), individual mouse (**f**) or independent culture replicates (**h**, **j**). Statistical significance (*p < 0.05, **p < 0.01, ***p < 0.01, ****p < 0.001) was drawn by two-tailed ordinary one-way ANOVA followed by Dunnett’s post-hoc test (**h**), two-tailed unpaired (**b**, **d**, **f**) and paired Student t-test (**j**)

Previous studies in AD brains have shown reduced expression of hnRNP A2 in neurons of the entorhinal cortex [32] but increased expression in the hippocampus [33]. However, modifications in hnRNP A2 expression in OLs in the context of AD are unknown. To characterize hnRNP A2 expression in AD, we classified human hippocampal tissues from controls and AD patients based on established neuropathological criteria. AD cases were defined by high Aβ burden (CERAD C and Thal phase 5), while controls showed minimal or no Aβ pathology [34, 35] (Supplementary Table S2). First, we performed Western blot analysis in order to quantifiy overall hnRNP A2 levels in the hippocampus. We showed a significant increased in hnRNP A2 expression in AD patients compared to controls (control 1.303 ± 0.34 *n* = 7; AD 4.439 ± 1.24 *n* = 6; arbitrary units, a.u.) (Fig. 2 a, b). Next, to determine whether this upregulation was specific to oligodendrocytes, hipppocampal tissues were immunostained for hnRNP A2 and Olig2, a specific marker for oligodendrocyte lineage cells. OLs exhibited increased levels of hnRNP A2 levels in AD patients compared to controls (control 44.64 ± 6.71 *n* = 3, 115 OLs; AD 55.90 ± 2.46 *n* = 4; 114 OLs, a.u.) (Fig. 2 c, d). In addition, Aβ (1 µM, 24 h) increased the hnRNP A2 levels in HOG cells, a human oligodedroglial cell line (control 0.475 ± 0.13; Aβ 0.625 ± 0.14, a.u., *n* = 4 independent cultures) (Supplementary Fig. S2a, b). These changes suggest a critical temporal dynamics of hnRNP A2 expression throughout the course of the disease in OLs that could be in part mediated by Aβ.

### hnRNP A2 expression is enhanced in OLs of mice models of AD and is dependent of Aβ

We then studied the expression dynamics of hnRNPA2 in the oligodendroglial population in the 3xTg-AD mouse hippocampus. OLs were identified using anti-Olig2,a marker that labels the entire oligodendroglial lineage. We observed a significant increase in hnRNPA2 expression in the nuclei of oligodendrocytes in the dentate gyrus of the hippocampus of 3xTg-AD mice compared to WT animals (WT 12.24 ± 1.64 vs. 3xTg-AD 27.22 ± 2.42, a.u., *n* = 7) (Fig. 2e, f). To determine whether hnRNP A2 could be also altered in neurons, we measured the mean intensity located in granule cells of dentate gyrus and observed a significant increase in the 3xTg-AD mouse compared to WT (WT 57.10 ± 6.83 vs. 3xTg-AD 120.5 ± 8.5, a.u., *n* = 3) ( Supplementary Fig. S2c, d). To elucidate the biological effect of Aβ on hnRNP A2 protein expression an in vivo model, we injected the peptide or vehicle into the hippocampus of C57BL6/J adult mice. Aβ injection (10 µM) led to an upregulation of hnRNP A2 protein in OLs in dentate gyrus, as revealed by fluorescence quantification in immunolabelled OLs (control 140.77 ± 9.01 vs. Aβ-injected 171.78 ± 8.36, a.u., *n* = 4) (Supplementary Fig. S2e, f).

To further describe the Aβ-induced modification on hnRNP A2 expression, rat primary oligodendrocyte cultures were treated with Aβ (1 µM, 3 h). We first observed a significant increase in the mRNA levels of hnRNP A2, as determined by RT-qPCR (control 2.06 ± 0.75 vs. Aβ 3.45 ± 0.57, a.u., *n* = 9) (Supplementary Fig. S2g). Western blot analysis confirmed a 44.5% ± 12.45 increase in hnRNP A2 protein levels after incubation with Aβ 1 µM for 24 h compared to controls (144.5% ± 12.45 vs. 100% of control, *n* = 7) (Fig. 2g, h). On the other hand, immunofluorescence analysis revealed that hnRNP A2 was abundantly present in the soma and processes of the oligodendroglial cells (Fig. 2i). Compared to control cells, Aβ treatment caused a significant increase in the granular pattern area in oligodendroglial soma (control 21.16 ± 2.03 vs. Aβ 26.95 ± 2.34, area, µm2, *n* = 6 and 60 OLs) (Fig. 2i, j). Overall, these findings showed that hnRNP A2 mRNA and protein overexpression in OLs is caused by Aβ in vitro and in vivo.

### Aβ remodels hnRNP A2 interactome in OLs

**Fig. 3:**
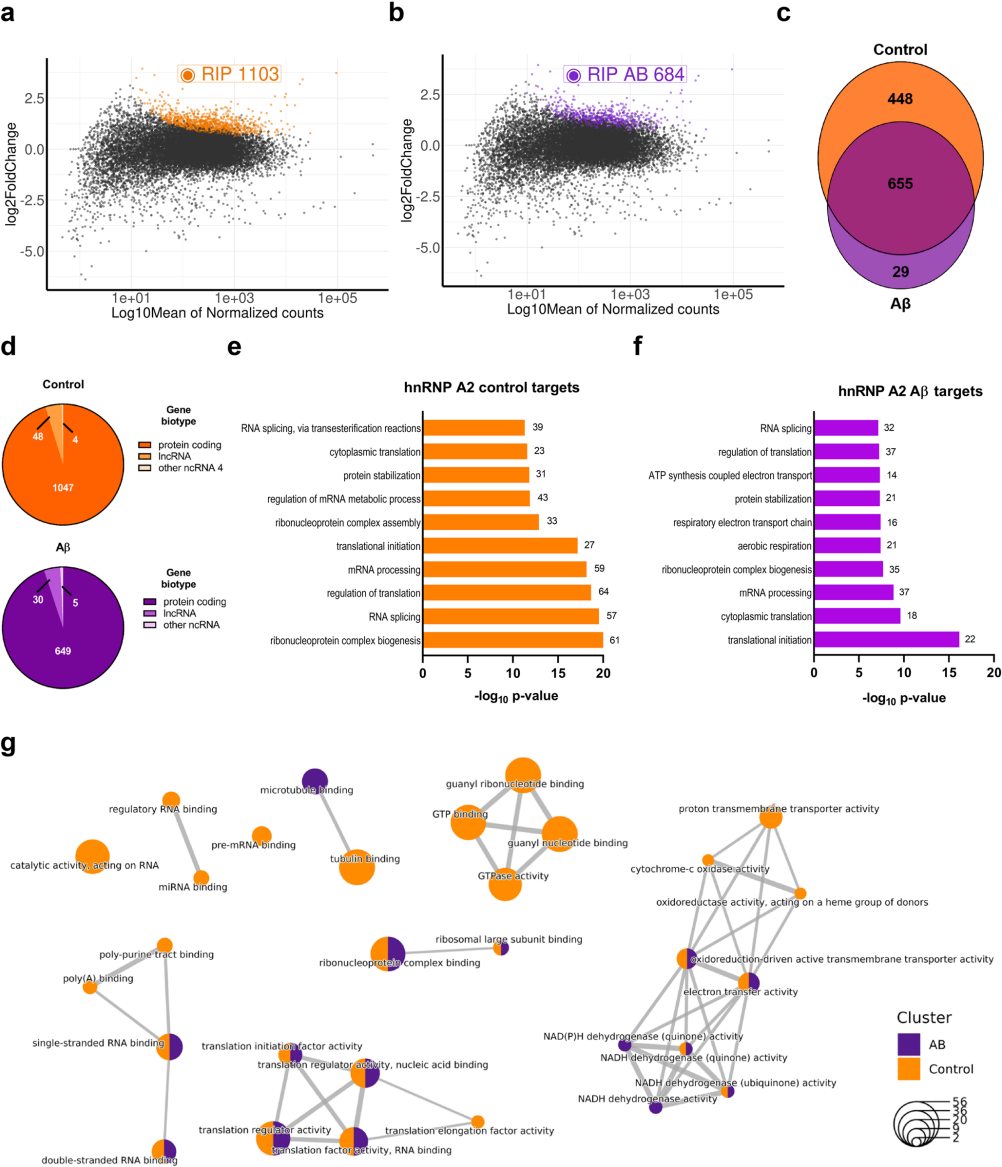
RIP-seq analysis of hnRNP A2-associated RNAs performed in primary cultured OLs. **a**, **b** MA plot of hnRNP A2 RIP-seq data in control and Aβ-treated cells. For each transcript, the average signal (measured as log10 mean of normalised counts) against the RIP-seq log2 Fold Enrichment (RIP versus IgG) was plotted. Significantly enriched targets are highlighted in orange (Ctrl) and purple (Aβ). **c** Venn diagram depicting the overlap between the hnRNP A2 interactome of control (orange) and Aβ-treated (purple) OLs. The numbers indicate the genes shared among the datasets. **d** Classification of hnRNP A2-associated RNAs in control (orange) and Aβ-treated (purple) OLs. The majority (95%) of identified targets are protein-coding genes, but long noncoding RNAs (lncRNAs) and other ncRNAs were also present. **e**, **f** Functional annotation of enriched hnRNP A2 target genes in control (orange) and Aβ-treated cells (purple). The barplot displays the top 10 enriched biological processes. The length of each bar is proportional to the statistical significance of the enrichment. The number of genes associated in each category is displayed beside the corresponding bar. **g** Network representation of the biological processes GO categories is enriched in the hnRNP A2 interactome in control (orange) and Aβ (purple) OLs. The size of the node indicates the number of genes in each GO term

HnRNP A2 is primarily localized to the nucleus where it regulates transcription initiation, promotes alternative splicing and facilitates the translocation of mRNAs from the nucleus to the cytoplasm [36]. HnRNP A2 exerts all these functions by binding to specific RNA sequences. These interactions of RBPs with their target RNAs are highly dynamic and allow the cell to respond to different environmental conditions. Despite the importance of this protein in health and disease, little is known about its associated transcriptome. To gain insight in the role of hnRNP A2 in OLs, we carried out RNA immunoprecipitation sequencing (RIP-seq) using anti-hnRNP A2 and isotype control antibodies (anti-IgG) in primary cultured OLs treated with Aβ (1 µM, 24 h, *n* = 3) (Supplementary Fig. S3a). Sequencing revealed 1,103 transcripts associated with hnRNP A2 in control OLs and 684 transcripts in Aβ-treated OLs (DeSeq2, Padj < 0.05; Fig. 3a, b, Supplementary Table S1). Notably, among these RNAs, 655 genes were common to both conditions and 29 were specific to Aβ-treated OLs (Fig. 3c). In both conditions, 95% were protein-coding transcripts, while 5% were long non-coding (lnRNAs) and 5% were other non-coding RNAs (ncRNAs) (Fig. 3d). To validate these target genes, our OL interactome was intersected with evidence from CLIP experiments in different human tissues and cell lines stored in POSTAR3 database [37]. In control conditions, 862 out of the 1,103 RNA targets were previously described to interact with hnRNP A/B family.

GO Term enrichment for biological processes (BPs) revealed that proteins coded by RNAs interacting with hnRNP A2 were significantly associated with ribonucleoprotein complex biogenesis, mRNA metabolism related processes and translational control (Fig. 3e). In Aβ-treated OLs, there was a decrease in the number of interacting genes, but there was not an overall switch in the associated GOs to these target genes (Fig. 3f). Of note, mRNAs encoding proteins involved in RNA-related metabolic processes and GTPases bound to hnRNP A2 were abundant in control OLs but absent in Aβ-treated OLs. Conversely, Aβ-specific interactors were associated more with microtubule binding and NADH dehydrogenase-related activity (Fig. 3g). Finally, we found hnRNPA2-associated transcripts, which encode components of RBP complexes, including *Hnrnpk*, *Hnrnpa1*, *Hnrnpf* and *Hnrnpa2b1* itself, suggesting an autoregulatory role. Importantly, in Aβ-treated OLs, the interactions between hnRNP A2 and Hnrnpk as well as its own mRNA were lost. These data support a differential regulation of the interactors of hnRNP A2 in Aβ treated OLs that may lead to its impaired function.

To explore a possible correlation between hnRNP A2-interacting genes altered in AD, we conducted a comparative analysis. Intriguingly, 357 genes (10.19% of 3,503) that interacted with hnRNP A2 were altered in both early and late-stage AD (Supplementary Fig. S3b). We then performed GO Term enrichment for the BPs, which revealed that among the most abundant interacting mRNAs, there were several factors involved in RNA-related processes (Supplementary Fig. S3c), consistent with our RIP-Seq results. Overall, these findings suggest that Aβ disrupts hnRNP A2’s functional network, potentially impairing RNA-related processes.

### Aβ alters the cargo of mRNA-hnRNP A2-containing granules and affects their activation state through the phosphorylation of hnRNP A2

Expression of MBP is a key step during oligodendrocyte maturation and hnRNP A2 is a central component of mRNA-containing cytoplasmic granules, binding directly to the A2RE in the 3′UTR of *Mbp* and *Mobp* mRNAs [38]. We previously demonstrated that Aβ oligomers upregulate local translation of *Mbp* through integrin β1 and Fyn kinase signalling [13]. This led us to investigate whether the upregulation of hnRNP A2 and its increase in the soma could affect the local translation of *Mbp* and *Mobp*.

**Fig. 4:**
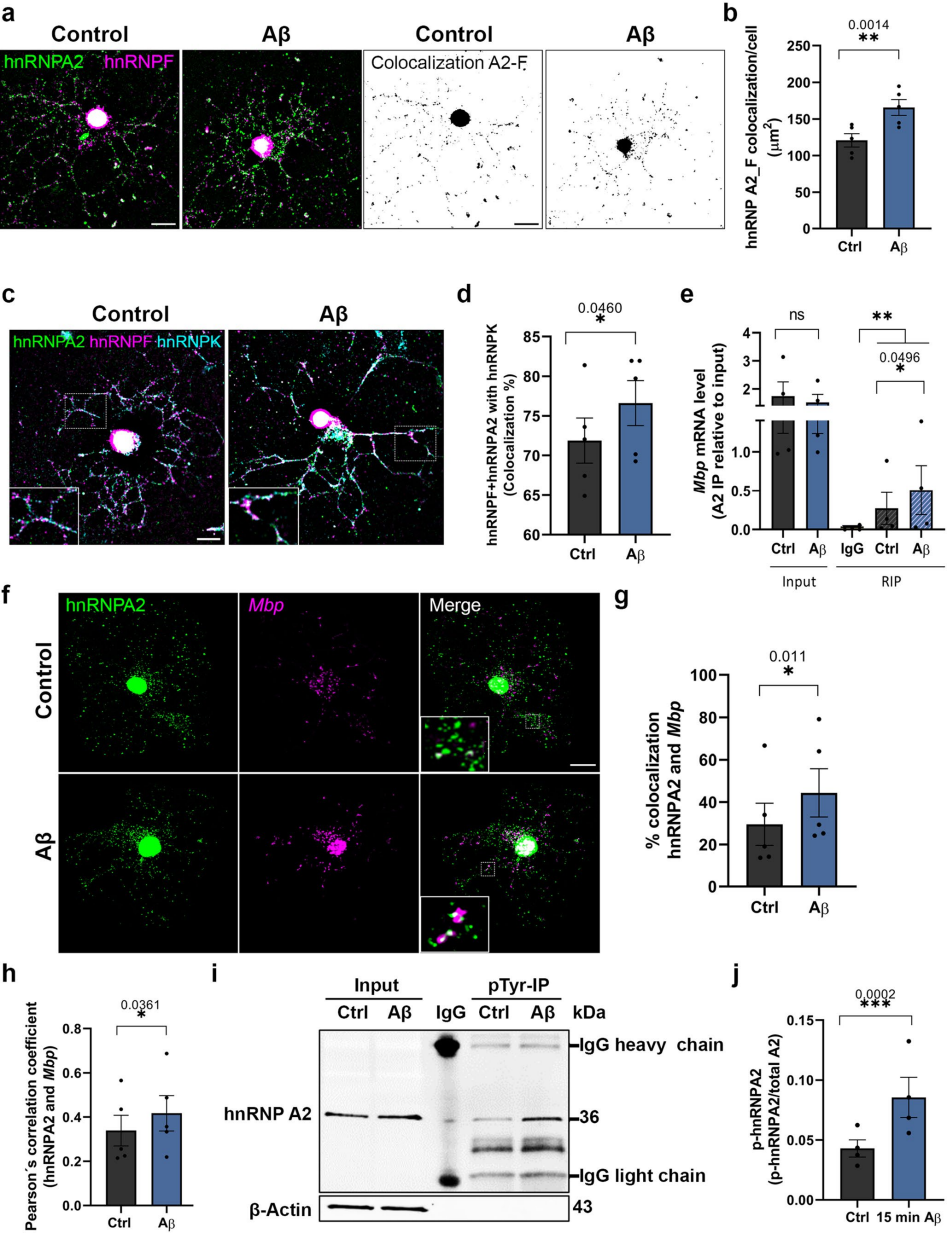
*Mbp* mRNA granule content and activation is affected by Aβ. **a**, **b** Double colocalisation confocal and binary images of hnRNP A2 (green) and hnRNP F (magenta). 1 µM Aβ significantly increased hnRNP A2-F colocalisation (µm2) (*n* = 5). Scale bar, 10 μm. **c**, **d** Triple colocalisation confocal images of hnRNP A2 (green), hnRNP F (magenta) and hnRNP K (cyan). Graphs show that 70% of all granules contain hnRNPK (active granules) and Aβ-treated OLs contain 5% more active granules (*n* = 5). Scale bar, 10 μm. **e** Analysis of *Mbp* mRNAs levels by RT-qPCR (*n* = 4). **f**, **g** Representative confocal images showing hnRNP A2 (magenta) and *Mbp* (green) transcript and the colocalized image. *Mbp* transcripts were found in the cytoplasm and nucleus of OLs. Aβ -treated cells show a higher percentage of colocalisation in the cytoplasm (*n* = 5). Scale bar, 10 μm. **h** Quantification of Pearson´s correlation coefficient for hnRNP A2 and *Mbp* (*n* = 5). **i**, **j** Western blot of pTyr-IP and IgG to detect hnRNP A2 phosphorylation. Histogram depicting the phosphorylation of hnRNP A2 normalized to total hnRNP A2 from input (*n* = 4). Data indicate the means ± S.E.M and dots represent independent culture replicates. Statistical significance (*p < 0.05, **p < 0.01, ***p < 0.001) was drawn by two-tailed paired Student´s t-test (**b**, **d**, **e**, **g**, **h**, **j**)

Since we observed increased hnRNP A2 expression following Aβ treatment, we first explored if Aβ might change the levels of other hnRNPs. To address this, we analysed both the mRNA and protein levels of various hnRNPs (hnRNP F, hnRNP E1, hnRNP K) typically found in *Mbp* and *Mobp* RNA granules. However, our analysis did not reveal any significant changes in either mRNA or protein levels for any of these hnRNPs (Supplementary Fig. S4a-c, *n* = 5). RNA granules are complex, dynamic structures that undergo multiple remodelling steps before mRNA translation can occur. As these granules reach the periphery, hnRNP E1 is replaced by hnRNP K, a crucial step for directing the mRNA to the myelin sheath and initiating translation (Supplementary Fig. S4d) [39]. Given our observations of hnRNP A2 upregulation, we asked whether these granules differ in terms of their composition. We quantified the co-localisation of hnRNP A2 and hnRNP F and observed a higher number of granules containing both components in Aβ-treated OLs (control 120.77 ± 9.09 vs. Aβ 165.58 ± 10.80, area, µm2, *n* = 5 and 50 cells) (Fig. 4a, b), suggesting an increase in granule number. Furthermore, Aβ-treated OLs demonstrated a notable increase in the total number of hnRNP A2-F-K active granules compared to control OLs (71.87% ± 2.845 vs. 76.60% ± 2.845, respectively; a.u., *n* = 5 and 50 OLs) (Fig. 4c, d).

To evaluate whether the increased number of granules also corresponds to a higher mRNA content, we examined the effect of Aβ on the interaction between hnRNP A2 and *Mbp* or *Mobp*. This was assessed using two distinct methodological approaches. First, we conducted RNA-immunoprecipitation (RIP) assay using anti-hnRNP A2 and its isotype control antibody (anti-IgG) (Supplementary Fig. S4f). Analysis of mRNA levels via RT-qPCR in the RIP fractions revealed a substantial enrichment of *Mbp* (control 0.21 ± 0.20 vs. Aβ 0.50 ± 0.31, *n* = 4) and *Mobp* (control 0.30 ± 0.14 vs. Aβ 0.62 ± 0.31, *n* = 4) mRNAs in Aβ-treated compared to control OLs, while no significant changes were observed in the input (*Mbp* control 1.74 ± 0.50 vs. Aβ 1.52 ± 0.28; *Mobp* control 1.57 ± 0.72 vs. Aβ 1.30 ± 0.46 (Fig. 4e, Supplementary Fig. S4g). Notably, we observed minimal expression of *Mbp* (0.017 ± 0.014) and *Mobp* (0.023 ± 0.012) in the control condition with the anti-IgG. Lastly, to determine if the enrichment was specific to *Mbp* and *Mobp*, we investigated Tau, which binds to hnRNP A2 [40]. No significant changes were observed in the RIP fractions (control 0.17 ± 0.14, Aβ 0.17 ± 0.13, and IgG 0.0014 ± 0.0006, *n* = 4) nor in the input (control 1.64 ± 0.19 vs. Aβ 2.01 ± 0.24) (Supplementary Fig. S4h). Second, we conducted fluorescent RNA in situ hybridization (FISH) assay by using probes to localize *Mbp* and *Mobp* mRNAs combined with inmunofluorescence for hnRNP A2 following Aβ treatment. Interestingly, colocalized area of hnRNP A2 with *Mbp* in the cytoplasm was 20.26 ± 4.72% in control OLs, and this increased significantly to 35.68 ± 9.48% in Aβ-treated OLs (*n* = 4, 39 cells) (Fig. 4f, g, Supplementary Fig. S5a, b) with no differences in the total area occupied by *Mbp* (Supplementary Fig. S5c). Similarly, for colocalizacion area of hnRNP A2 with *Mobp* was a 12.52 ± 0.59% in control OLs while in Aβ-treated OLs was 19.97 ± 0.59% (*n* = 4, 39 cells) (Supplementary Fig. S5d, e, g, h ) with no differences in the total area occupied by *Mobp* (Supplementary Fig. S5f). Additionally, Pearson colocalization coefficient was also higher in Aβ-treated OLs for *Mbp* (Ctrl 0.338 ± 0.15 vs. Aβ 0.416 ± 0.17, *n* = 5) and *Mobp* (Ctrl 0.252 ± 0.02 vs. Aβ 0.336 ± 0.02, *n* = 4) (Fig. 4h, Supplementary Fig. S5i). These experiments indicated that Aβ promotes *Mbp*- and *Mobp*-containing hnRNP A2 granules in OLs.

To initiate translation, hnRNPA2 must be disassembled from the mRNA granule through phosphorylation of tyrosine residues by the tyrosine protein kinase Fyn [41]. Therefore, to determine if Aβ increases hnRNP A2 phosphorylation, we conducted an immunoprecipitation assay using an anti-pTyr (PY99) antibody (Fig. 4i), followed by a Western blot with hnRNP A2. Data demonstrated that after Aβ treatment (1 µM, 15 min), the levels of phosphorylated hnRNP A2 were significantly higher than those observed in the control (Fig. 4j) (0.042 ± 0.007 vs. 0.085 ± 0.016, *n* = 4, a.u., respectively). These findings suggest that Aβ-induced alterations in hnRNP A2 dynamics and phosphorylation could drive localized synthesis of *Mbp* and *Mobp* mRNAs.

### Aβ enhances the local synthesis of *Mbp* and *Mobp* in an hnRNP A2-dependent manner

**Fig. 5:**
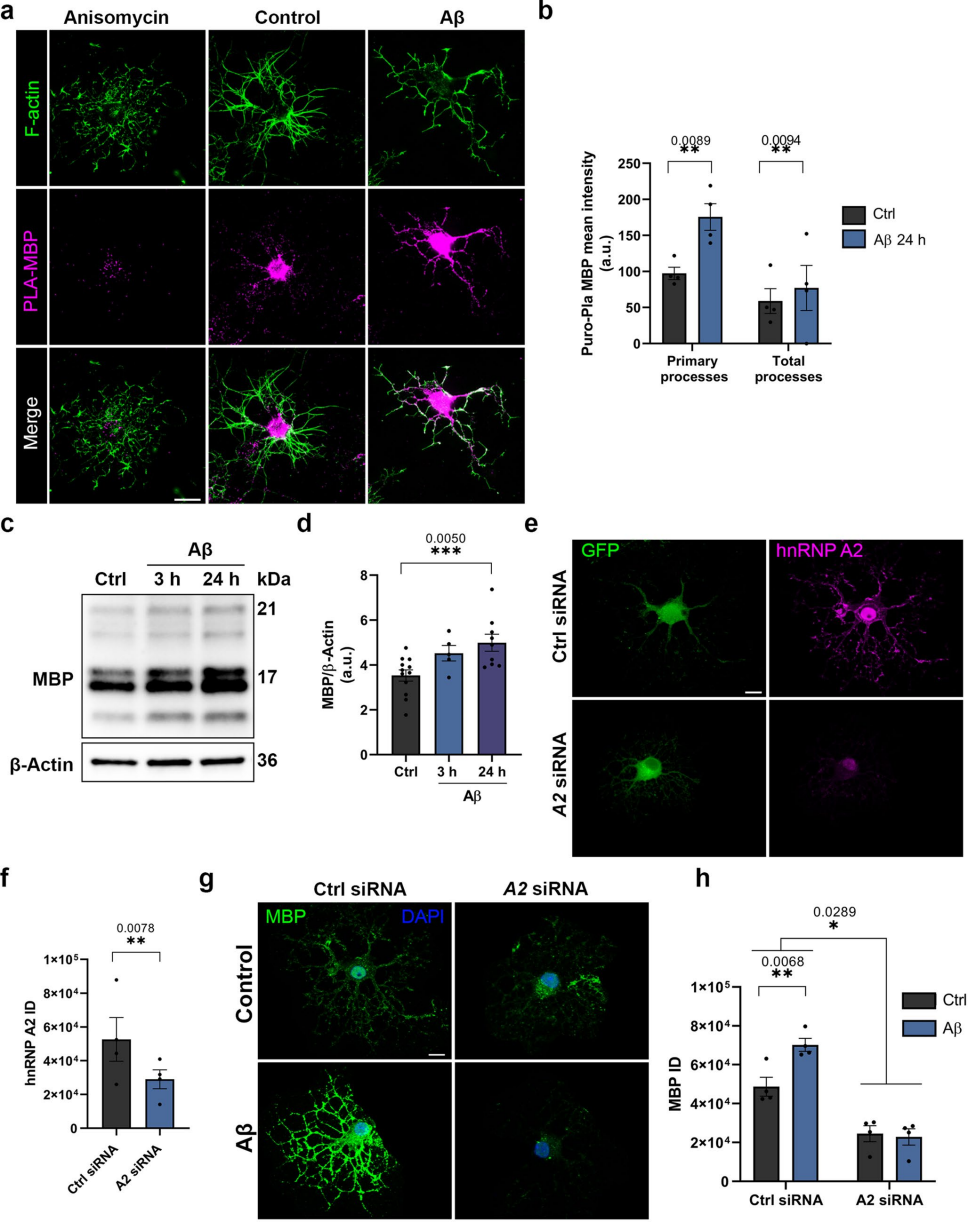
Regulation of *Mbp* synthesis by Aβ is dependent of hnRNP A2. **a**, **b** Photographs show MBP puro-PLA-positive puncta in the soma, primary and total processes. MBP PLA positive puncta was analysed in bins of 10 μm ranging from the soma in primary and total processes (*n* = 4). Scale bar, 10 μm. **c**, **d** MBP expression and relative quantification in oligodendrocyte cell extracts normalised to β-actin (*n* ≥ 4). **e**, **f** Representative confocal micrographs of GFP (green) and hnRNP A2 (magenta) in OLs transfected with Ctrl or *Hnrnpa2*-targeting siRNAs. Histogram depicting the hnRNP A2 integrated density (ID) within OLs (*n* = 4). Scale bar, 10 μm. **g**, **h** Representative confocal micrographs of MBP (green) and DAPI (blue) in untreated and treated OLs transfected with Ctrl or *Hnrnpa2*-targeting siRNAs. Scale bar, 10 μm. Histogram depicting the MBP integrated density (ID) within OLs (*n* = 4). Data are represented as means ± S.E.M and dots indicate independent culture replicates. Statistical significance (*p < 0.05, **p < 0.01, ***p < 0.001) was drawn by two-tailed paired Student´s t-test (**b**, **f**), one-way ANOVA followed by Dunnett’s post-hoc test (**d**) and two-way ANOVA followed by Sidak´s post-hoc test (**h**)

To investigate whether the observed changes promote the synthesis of *Mbp* and *Mobp* mRNA, we used puromycin together with proximity ligation assay (Puro-PLA) to assess translation. To visualise oligodendrocyte processes, the cytoskeleton was labelled with phalloidin, which binds to F-actin. We quantified the PLA intensity in both primary processes (those emerging from the soma) and total processes to determine if there were differences in localization (Fig. 5a). Our findings revealed that Aβ-treated OLs exhibited significantly enhanced local translation of *Mbp*, both in primary processes (control 162.50 ± 63.66 vs. Aβ 262.80 ± 76.43, a.u., *n* = 4) and total processes (control 54.34 ± 18.56 vs. Aβ 89.74 ± 21.96, a.u., *n* = 4) (Fig. 5a, b). However, the results for *Mobp* local translation were less definitive. While Aβ induced increased local synthesis of MOBP in primary processes (control 6.43 ± 2.84 vs. Aβ 140.35 ± 104.04, a.u., *n* = 3), no significant difference was observed in total processes (control 42.60 ± 27.28 vs. Aβ 55.55 ± 37.35, a.u., *n* = 3) (Supplementary Fig. S6a, b). To confirm the translation dependency of the observed puncta in puro-PLA images, we included anisomycin as a negative control. As depicted in the puro-PLA images, OLs preincubated with anisomycin did not exhibit any PLA-positive puncta, confirming that the puncta we observed in puro-PLA were indeed translation dependent. We further confirmed these results by Western blot analysis by analysing MBP and MOBP total protein levels in OLs lysates (Fig. 5c, d and Supplementary Fig. S6c, d), in the hippocampus of 6-month-old 3xTg-AD mice (WT 0.7093 ± 0.05 *n* = 6 vs. 3xTg-AD 1.032 ± 0.22, *n* = 5, a.u.) (Supplementary Fig. S6e, f) [13], and in human hippocampus (Control 262.9 ± 67.01 *n* = 5 vs. AD 381.4 ± 78.43, *n* = 6, a.u.) (Supplementary Fig. S6g, h). Interestingly, a low molecular weight band was detected in the MBP Western blot from human hippocampal tissue in three AD patients and three control individuals. This band was not consistently present across all samples and may suggest the presence of a degraded form of MBP, potentially reflecting alterations in myelin integrity or turnover in a subset of individuals.

To see if Aβ-triggered MBP overexpression was mediated by hnRNP A2, the hnRNP A2 gene was knocked down with a small interfering RNAs (siRNAs) and MBP expression was measured by immunofluorescence assay. The *Hnrnpa2b1*-targeting siRNA decreased hnRNP A2 expression 42.89% ± 5.30 compared to control (control expression, 100%, *n* = 4) (Fig. 5e, f). Immunofluorescence analysis revealed a significant increase of MBP in Aβ-treated OLs, which was reverted in by silencing of *Hnrnpa2b1* (Ctrl siRNA control 48,703 ± 4,87 vs. Aβ 70,189 ± 3.32, *A2* siRNA control 24,500 ± 4,148 vs. Aβ 22,844 ± 4,206, a.u., *n* = 4) (Fig. 5g, h). In summary, these results collectively suggest that modifications in the hnRNPA2 protein by Aβ enhances the MBP and MOBP local synthesis in OLs.

### Aβ-induced MBP overexpression reduces the voltage-gated calcium influx in OLs

**Fig. 6:**
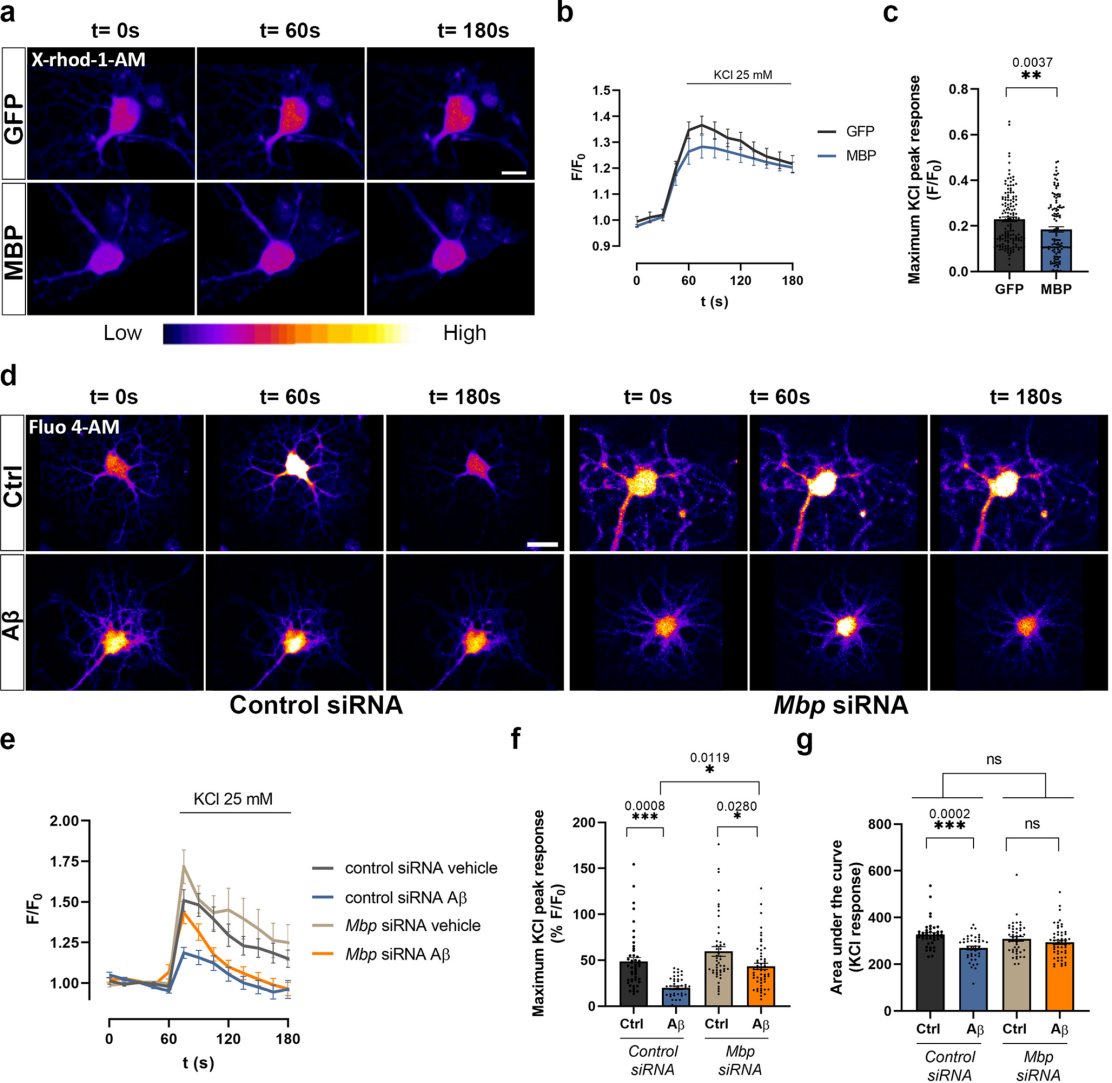
MBP overexpression inhibits KCl-induced Ca^2+^ influx into OLs. **a** OLs infected with either AAV8-pMBP-GFP or AAV8-pMBP-MBP-IRES-GFP were loaded with x-Rhod-1 AM. Time course of intracellular Ca^2+^ levels were recorded before and after KCl 25 mM stimulus by confocal microscopy (*n* = 7). Scale bar, 10 μm. **b**, **c** Graphs show the maximum peak of KCl response. **d** OLs transfected with either control siRNA or *Mbp*-targeting siRNAs were loaded with Fluo-4 AM and exposed to 1 µM Aβ for 24 h. Time course of intracellular Ca^2+^ levels were recorded before and after KCl 25 mM stimulus by confocal microscopy (*n* = 6). Scale bar, 20 μm. **e-g** Graphs show the maximum peak and the area under the curve (AUC) of KCl response in the different conditions. Data indicate means ± S.E.M and dots represent individual cells. Statistical significance (*p < 0.05, **p < 0.01, ***p < 0.001, ****p < 0.0001) was drawn by two-tailed unpaired Student´s t-test (**c**) and ordinary two-way ANOVA followed by Sidak´s post-hoc test (**f**, **g**)

MBP regulates voltage-gated Ca^2+^ channels (VGCCs) at the plasma membrane reducing the Ca^2+^ influx in the oligodendrocyte cell line N19 as well as in primary cultures of oligodendroglial progenitor cells [42]. To assess whether MBP levels could modify Ca^2+^ homeostasis in mature OLs, an AAV8-mediated MBP overexpression (AAV8-pMBP-MBP-IRES-GFP) was performed in OLs in vitro (Supplementary Fig. S7a, b). Ca^2+^ recordings after application of KCl 25 mM to OLs loaded with X-rhod-1 showed a reduced Ca^2+^ influx in cells overexpressing MBP with respect to control cells (AAV8-pMBP-GFP) (Ctrl 0.2294 ± 0.01 vs. Aβ 0.1845 ± 0.01, *n* = 7, 140 and 123 OLs respectively) (Fig. 6a-c).

Next, we knocked down MBP expression in OLs by using siRNAs against *Mbp* and a non-targeting siRNA as a negative control. Initially, we validated the reduction in *Mbp* gene expression using Western blot analysis of 21, 17 and 18 kDa MBP isoforms (Supplementary Fig. S7c). The *Mbp*-targeting siRNA decreased the 21 kDa isoform 38.78% ± 10.14 and the 17 and 18 kDa isoforms 19.49% ± 10.50 compared to the negative control (control expression, 100%, *n* = 6). In addition, Aβ-treated OLs exhibited an increase in MBP 21 kDa (121.6% ± 7.78) and 17–18 kDa isoforms (132.1% ± 11.92), an effect that was abolished when MBP was experimentally reduced (Supplementary Fig. S7d).

To study whether Aβ-induced MBP overexpression can dysregulate Ca^2+^ homeostasis, we performed Ca^2+^ imaging experiments in OLs with siRNAs targeting and non-targeting *Mbp*. First, we observed reduced levels of resting intracellular Ca^2+^ in *Mbp* knockdown OLs compared to controls (Supplementary Fig. S7f). Also, Ca^2+^ influx after plasma membrane depolarization with KCl was reduced in Aβ-treated OLs compared to control ones (0.448 ± 0.043 vs. 0.2091 ± 0.024, *n* = 6 and 47 OLs and *n* = 6 and 41 OLs), which was partially restored by silencing MBP (0.5967 ± 0.056 vs. 0.3966 ± 0.032, *n* = 6 and 47 cells and *n* = 6 and 55 OLs) (Fig. 6d-f). Moreover, we observed a decrease in the area under the curve in Aβ-treated OLs (313.7 ± 5.49 vs. 272.8 ± 7.49), which was fully recovered when MBP was reduced (300.7 ± 8.37 vs. 289.1 ± 8.36) (Fig. 6g). Finally, to investigate whether hnRNP A2 plays a role in the Ca^2+^ homeostasis, Ca^2+^ levels were measured after 25 mM KCl stimulation in OLs treated or not with Aβ, with and without siRNA-mediated knockdown of *Hnrnpa2b1* expression. (Supplementary Fig. S7g). The results showed that Aβ treatment led to a decrease in Ca^2+^ influx (0.521 ± 0.056 vs. 0.357 ± 0.029, *n* = 3, 28 OLs and 21 OLs) (Supplementary Fig. S7h, i); however, this effect was absent when *Hnrnpa2b1* expression was reduced (0.4613 ± 0.048 vs. 0.3767 ± 0.024, *n* = 3, 22 OLs and 23 OLs), suggesting that hnRNP A2 is required for the modification of Aβ-induced Ca^2+^ dynamics. Interestingly, analysis of the AUC revealed that the knockdown of *Hnrnpa2b1* expression alone also alter Ca^2+^ levels (Ctrl siRNA Ctrl 27.98 ± 1.45 vs. Ctrl siRNA *A2* 24.96 ± 0,44, *n* = 3, 28 OLs and 23 OLs), indicating that hnRNP A2 by itself may influence Ca^2+^ homeostasis, independent of Aβ treatment (Supplementary Fig. S7j). Taking together, these findings indicate that MBP overexpression inhibits Ca^2+^ influx through VGCCs, suggesting that Aβ plays a role on Ca^2+^ dynamics by modulating MBP levels.

## Discussion

Emerging evidence highlights the significant role of WM degeneration in the pathology of AD, although the exact causes remain under investigation. Abnormalities in myelin and OLs in AD are associated with elevated levels of Aβ peptides [8]. These changes occur before neuronal damage, and OL loss may contribute to cognitive deficits [10].

Common perturbations in RNA metabolism appear in several neurodegenerative diseases, standing out the critical importance of RBP homeostasis in brain physiology [29]. In this study, we conducted a comprehensive approach including immunohistological and protein expression analysis of post-mortem human brains, bulk and hnRNP A2-RIP transcriptomics of Aβ-treated primary cultured OLs, and functional analysis to monitor myelin protein translation and intracellular Ca^2+^ levels. We initially reported elevated levels of hnRNP A2 in human hippocampal OLs of AD patients, as well as in AD mice brains. Subsequently, we systematically investigated the impact of Aβ-induced hnRNP A2 dysregulation in OLs from AD models. Our study demonstrated that Aβ alters the hnRNP A2 interactome, affecting mRNA associations, cargo, and RNA granule trafficking. These alterations caused to changes in the local translation of key myelin proteins, MBP and MOBP, which subsequently disrupted oligodendroglial Ca²⁺ homeostasis. Although most of the work was carried out in cultured OLs, these findings collectively suggest changes for OLs maturation, survival, and the maintenance of myelin integrity all of which could contribute to the pathogenesis and progression of AD.

OLs show functional alterations in AD, including myelination, neuronal activity sensing and immune responses [43]. Furthermore, the proteomic profile of AD brain networks has revealed a consistent upregulation of oligodendroglia-enriched modules associated with myelination [44, 45], suggesting their active involvement in AD pathology. The signalling of Aβ peptide affects the transcriptional profiles of OLs, with spatial transcriptomic analyses showing distinct transcriptomic responses within plaque environments, suggesting that soluble Aβ peptide alters the OL response during the disease progression [46]. Notably, recent studies demonstrate that OLs themselves produce Aβ alongside neurons, as observed in AD mouse models [9, 47] and human post-mortem brains [48]. In our study, conducted in vitro using primary cultured OLs, we further show that Aβ peptides remodel the OL transcriptome, particularly genes related to hnRNPs and RNA metabolism, such as *App*, *Hnrnpa2b1*, *Hnrnpf*, *Hnrnpm*, and *Hnrnpu*. In agreement with these results, proteomic analysis have shown upregulated modules in AD related with RNA binding and splicing proteins [44] with hnRNP A2 emerging as a common hub [45].

RBPs play a crucial role in RNA metabolism processes by forming complexes that regulate pre-mRNA splicing, transcription, and translation. Amongst the most abundant RBPs are the members of hnRNP A/B family, namely hnRNP A1 and A2/B1. To date, most studies examining hnRNP A2 expression have focused on neurons, with findings showing variability across brain regions and disease stages. For example, Mizukami et al. reported increased hnRNP A2 levels in the CA2 region of patients with mild cognitive impairment (MCI) [32], whereas other studies have observed decreased expression in the hippocampus of MCI and AD patients [33, 49]. In our study, we found increased hnRNP A2 levels specifically in OLs within the hippocampus of AD patients with high Aβ burden, as well as in 3xTg-AD mouse model. Importantly, depletion of hnRNP A2 led to the production of a more active β-secretase isoform (BACE1), which may contribute to the accumulation of Aβ plaques [50]. Therefore, hnRNP A2 exhibits region- and stage-specific changes in AD, linking its dysregulation to altered molecular mechanisms, including increased β-secretase activity and Aβ plaque formation. Additionally, the dysregulation of RBPs in OLs may disrupt RNA metabolism and myelin protein translation, further contributing to AD pathology.

This study provides the first comprehensive identification of transcripts associated with hnRNP A2 in OLs. HnRNP A2 regulates genes critical for RNA biology, including those involved in RNA metabolism, processing, and splicing, as well as the biogenesis of ribonucleoprotein complexes and translational regulation. Notably, we found that Aβ upregulates hnRNP A2 and remodels its transcriptomic associations, weakening its interaction with over 50% of total mRNAs, potentially causing alternative splicing or mRNA decay [36]. Aβ-induced alterations in hnRNP A2 interactions may, therefore, have profound downstream effects on the transcriptome and proteome of OLs, with implications for myelin integrity. Aβ specifically disrupts hnRNP A2 binding to RNAs involved in GTPase signalling, critical for OL survival and myelination [51], while enhancing its binding to RNAs linked to microtubule dynamics and OXPHOS, suggesting effects on cytoskeletal organization and energy metabolism.

Additionally, in response to Aβ peptide, oligodendrocyte modify the translation of MBP and MOBP, along with functional alterations in Ca^2+^ homeostasis, which in turn arise from the dysregulation of hnRNP A2. Specifically, these features may result from the convergence of two distinct mechanisms. The first increases the availability of granules enriched with *Mbp* and *Mobp* mRNA. The second involves the activation of Fyn kinase by Aβ [13] leading to the phosphorylation of hnRNP A2. In the present study, we provide the first evidence that Aβ promotes local translation of *Mbp* and *Mobp* by altering the cargo, number and dynamics of mRNA granules through regulation of hnRNP A2. We found a higher association between hnRNP A2 and *Mbp* and *Mobp* mRNAs, a significant increase in the number of granules containing hnRNP A2 and F, as well as active granules containing hnRNP K.

MBP is critical for myelin integrity, with its absence leading to myelin vesiculation and subsequent breakdown [52]. In our study, we report a significant increase in MBP levels following Aβ treatment in cultured OLs, in the hippocampus of early-stage 3xTg AD mice, and in human hippocampal lysates from individuals with high Aβ levels. Additionally, in our recent work using a zebrafish model, we showed that in vivo exposure to Aβ leads to increased expression of myelin-related proteins, including MBP, during developmental stages, further supporting the hypothesis that soluble Aβ species can stimulate myelination under certain conditions [53]. These findings suggest that MBP expression is upregulated in response to Aβ exposure, possibly as part of a compensatory OL response during the progression of AD. However, the role of MBP in AD pathophysiology remains complex and somewhat controversial. While several studies have reported increased MBP levels in AD brains [54], others have found reduced MBP levels, particularly in the WM of AD patients [55]. Interestingly, MBP has been shown to degrade Aβ in vitro [56], suggesting a possible protective role. Conversely, degraded MBP has also been found around Aβ plaques, suggesting localised myelin damage in the AD brain [54]. In addition, clinical studies using cerebrospinal fluid (CSF) have shown that MBP levels follow a non-linear trajectory with age initially increasing, then decreasing, and rising again in late adulthood. Elevated CSF MBP levels have been associated with worse cognitive scores, more tau pathology, and higher levels in Aβ + individuals and APOE-ε4 carriers [57]. Taking together, these data suggest a biphasic pattern of MBP regulation in AD: an early upregulation that may reflect a compensatory response to Aβ-induced myelin stress and a later decline or degradation resulting from persistent OL dysfunction, neuroinflammation, and disease progression. Understanding the temporal and context-specific roles of MBP may be crucial for designing therapies aimed at preserving myelin integrity and modulating glial responses in AD.

Upregulation of MBP inhibits VGCCs, thereby reducing Ca^2+^ influx into OLs [42]. Ca^2+^ influx through membrane channels is a critical step in signalling pathways involved in the regulation of growth, maturation and functional plasticity. In addition, elevated Ca^2+^ levels stimulate MBP synthesis in OPCs [58] and are critical for myelin sheath extension by remodelling the actin cytoskeleton [59]. Recent study show that spontaneous localized Ca^2+^ transients, driven by store-operated entry and internal stores are crucial for OL process branching and myelin sheet formation. Disruption of these transients significantly impairs morphological maturation [60]. Moreover, OLs integrate distinct Ca^2+^ signatures across development, with transient, localized Ca^2+^ spikes playing a key role in myelin segment growth and axonal wrapping. The precise Ca^2+^ regulation is not only necessary for differentiation but also for activity-dependent myelination, suggesting that any persistent disruption in Ca^2+^ signalling, such as that caused by Aβ, could significantly impair myelin formation and repair mechanisms [61]. Here, we demonstrate that Aβ inhibits the Ca^2+^ influx into the cell through VGCCs, which is partially recovered by siRNA-mediated MBP downregulation. Since Ca^2+^ entry stimulates MBP synthesis and MBP in turn inhibits Ca^2+^ entry, our results may suggest a self-regulatory mechanism in which MBP inhibits VGCCs to regulate its own synthesis. The accumulation of MBP in the membrane would inhibit or reduce the number of VGCCs in the membrane, as previously reported [42], thereby affecting to the Ca^2+^ entry through these channels. Thus, our results suggest that Aβ peptide, in a MBP-dependent manner, disrupt Ca^2+^ regulation, making OLs more susceptible to environmental stimuli that increase intracellular Ca^2+^ levels and potentially impairing myelin formation and repair. However, these results are primarily drawn from controlled cell culture model using inmature and mature OLs, where Ca^2+^ dynamics have been shown to vary significantly dependeding on the maturation state [62, 63]. Therefore, further validation in more complex in vivo systems, such as by assesing calcium dynamics in OLs of AD mouse models, is needed to confirm these findings.

Overall, this study reinforces the importance of OLs in AD, not only by discovering variations in RNA dynamics and the role of hnRNP A2, but also by identifying novel mechanisms implicating MBP and Ca^2+^ in AD pathophysiology. While much of the work was conducted in vitro, the relevance of these findings is supported by complementary observations in 3xTg-AD mouse models and human post-mortem samples.

## Electronic supplementary material

Below is the link to the electronic supplementary material.

Supplementary Material 1 (DOCX 9.0 MB)

Supplementary Material 2 (XLSX 401 KB)

Supplementary Material 3 (XLSX 229 KB)

## Data Availability

The data used in this study is available on the GEO repository with the identifier GEO GSE263799.

## Abbreviations

AD: Alzheimer´s disease
OLs: Oligodendrocytes
MBP: Myelin basic protein
Aβ: Amyloid-β peptide
hnRNPA2: Heterogeneous nuclear ribonucleoprotein A2/B1
WM: White matter
CNS: Central nervous system
RBP: Ribonucleoprotein
MCI: Mild cognitive impairtment
CSF: Cerebrospinal fluid
