# Supplementary material for: Disruption of hnRNP A2-mediated RNA dynamics by amyloid-β drives MBP increase in Alzheimer’s disease
Source: Cell Mol Life Sci. 2025 Aug 2;82(1):298. doi: 10.1007/s00018-025-05823-5 (PMC12317959; doi:10.1007/s00018-025-05823-5)
Supplement: Supplementary file 1 — (DOCX 9.0 MB) [file 18_2025_5823_MOESM1_ESM.docx]

**SUPPLEMENTARY FIGURES AND TABLES**


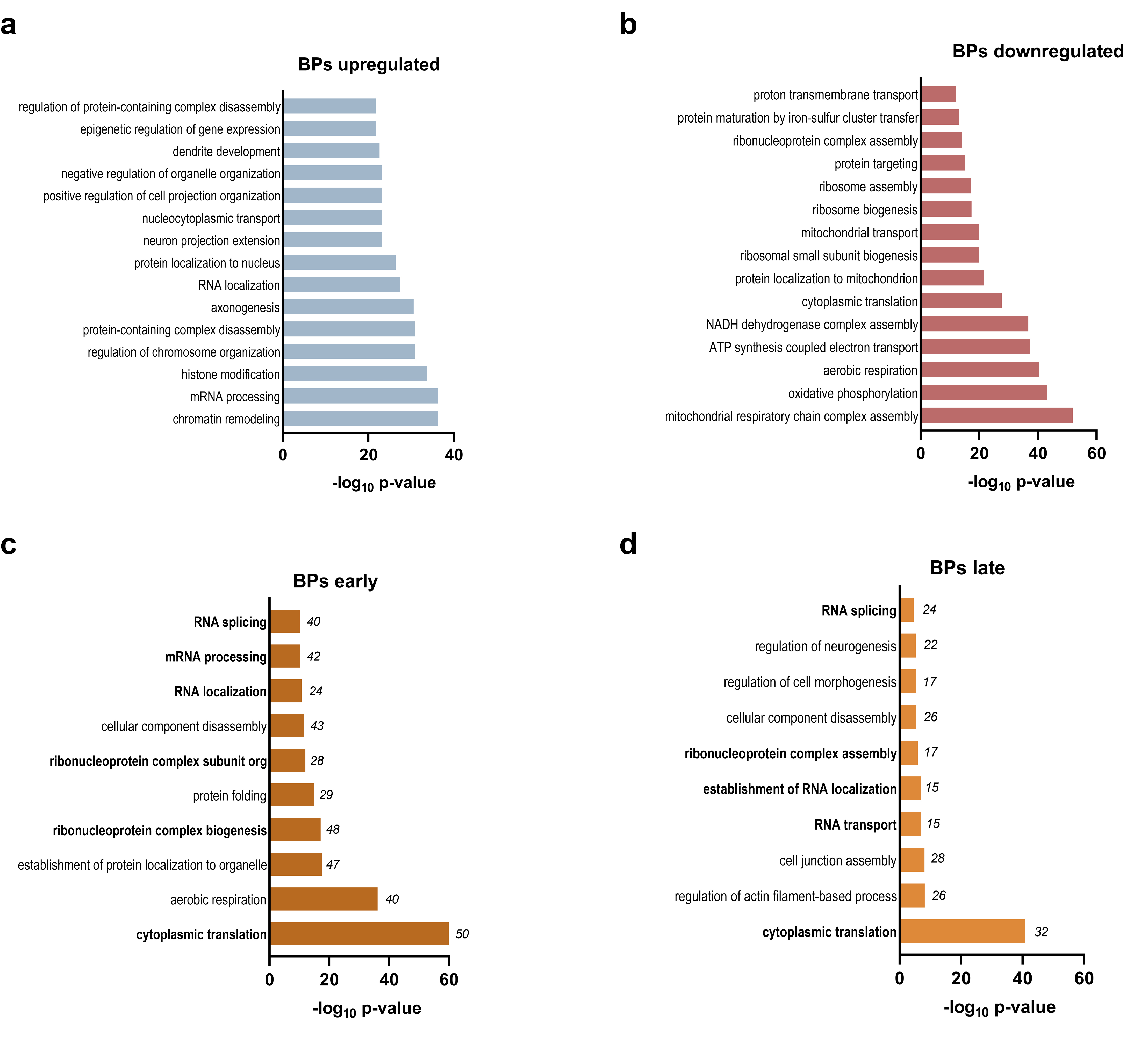


**Figure S1. Analysis of functional transcriptional changes in OLs in AD.** (**a, b**) Gene ontology enrichment of Biological Process (BP) of upregulated genes (a, blue) and downregulated genes (b, red) in vitro after 24 hours of 1µM of Aβ treatment. (**c, d**) Gene ontology enrichment analysis (BP only) shared in our Aβ-treated OLs and early- (c) and late- (d) AD conditions [28].


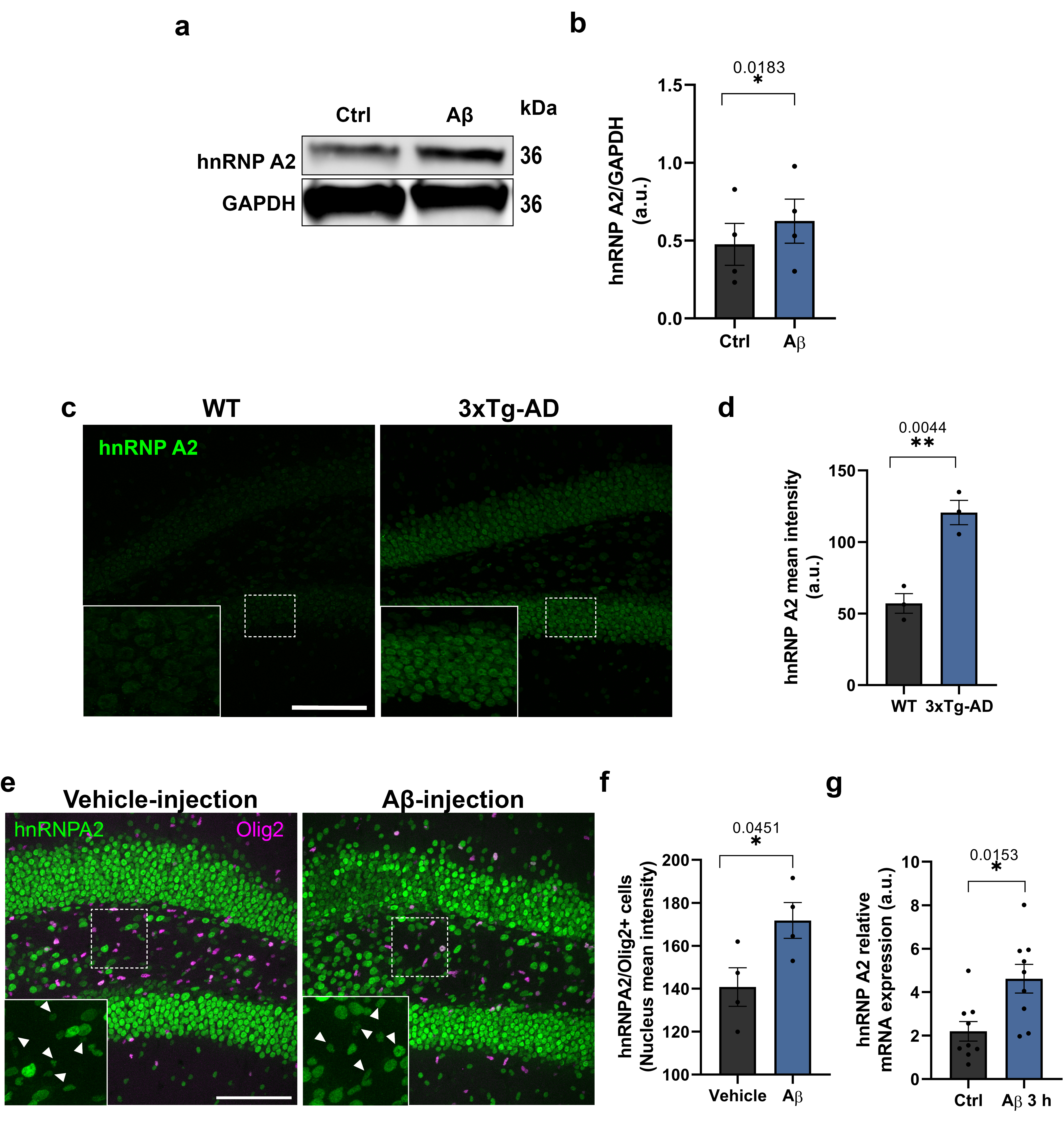


**Figure S2. Analysis of hnRNP A2 expression in different models.** (**a, b**) hnRNP A2 expression and relative quantification in HOG cell extracts treated and untreated with 1 µM of Aβ (n=4). (**c, d**) Representative confocal images of hnRNP A2 (green) in the dentate gyrus of WT and 3xTg-AD mice. Quantification of hnRNP A2 mean intensity signal in granular neurons in the dentate gyrus (n=3). Scale bar, 100 µm. (**e, f**) Representative confocal images of Olig2 (magenta) and hnRNP A2 (green) in the dentate gyrus of vehicle (n=4) and Aβ-injected mice (n=4). Quantification of hnRNP A2 intensity in Olig2^+^ cells in the dentate gyrus. Scale bar, 100 µm. (**g**) RT-qPCR analysis of *Hnrnpa2b1* mRNA expression in Aβ-treated and control OLs (n=9). Data indicate means ± S.E.M and dots represent independent culture replicates (**b, g**) or individual animals (**d, f**). Statistical significance (*p<0.05, **p<0.01, ***p<0.001) was drawn by two-tailed paired Student t-test (**b, g**) and by unpaired Student t-test (**d, f**).


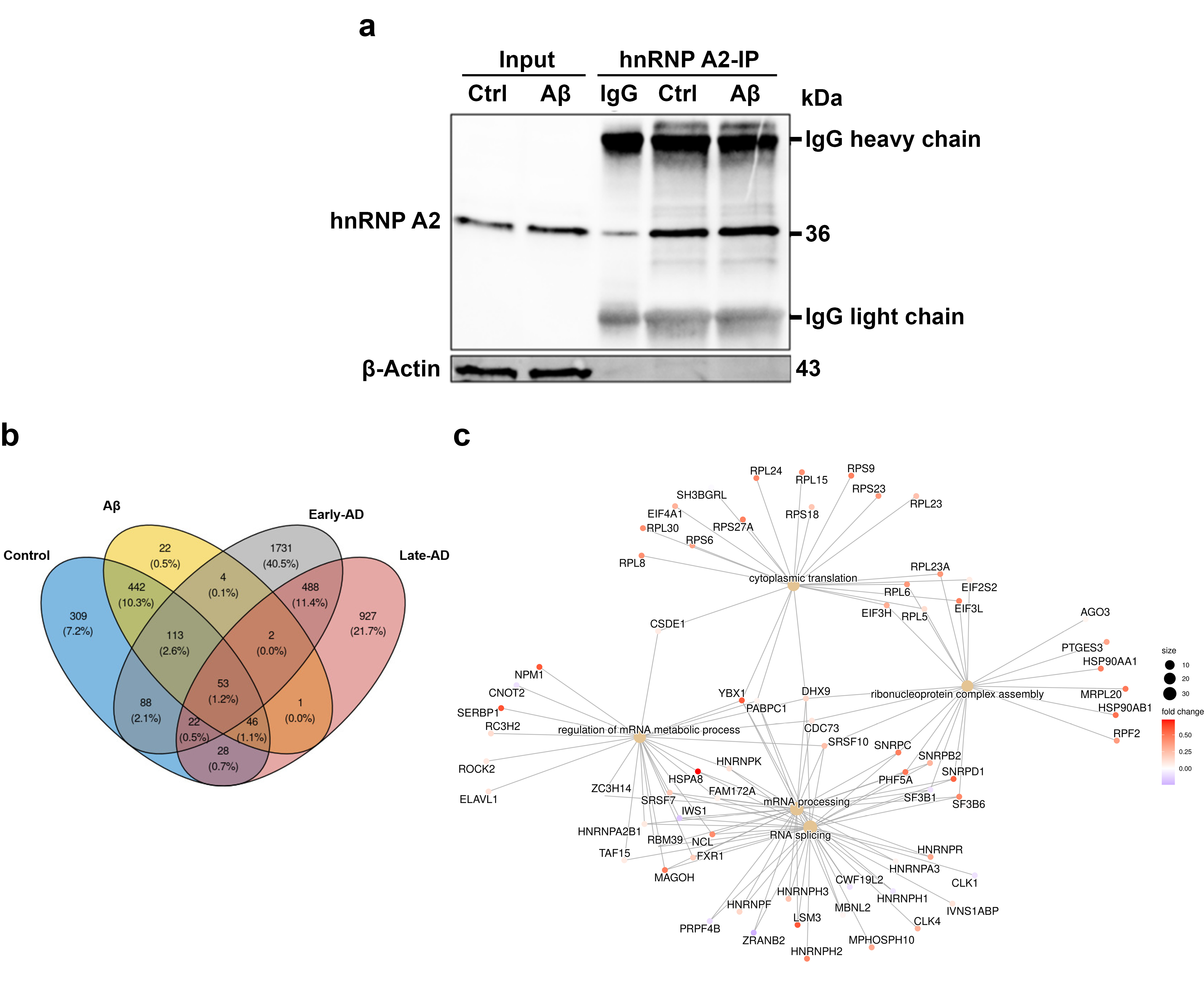


**Figure S3. RIP-seq analysis of hnRNP A2 interactome performed in primary cultured OLs compared to human gene signatures of AD.** (**a**) IP was performed using hnRNP A2 antibody-coated agarose beads. Negative control of isotype IgG was used to detect non-specific binding of proteins to antibody. Proteins were eluted and loaded in 10% SDS-PAGE gel for analysis by western blot. (**b**) Venn diagram depicting the overlap between the hnRNP A2 interactome of control and Aβ-treated OLs and the DEGs in human early and late-AD patients [19]. Percentage and numbers indicate the genes shared among the conditions. (**c**) Top 5 GOs CNETPlot. Network visualization of DEGs involved in the top 5 enriched GO terms. Each gene is linked with its respective GO term or terms, if shared. The fold change (from early-AD) is shown colour and the number of differentially expressed genes per term is shown in size for each node.


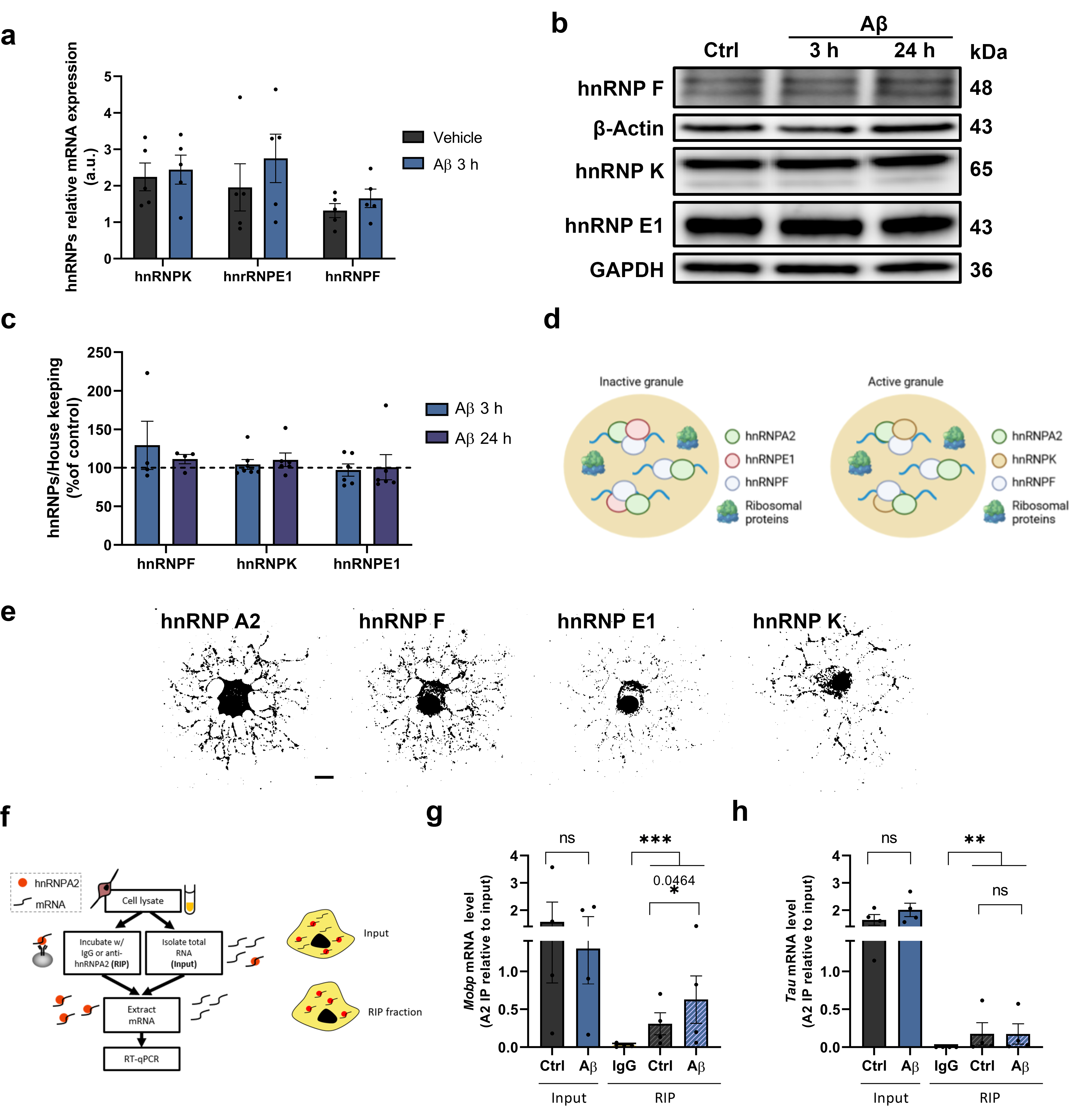


**Figure S4. mRNA granule description and dynamic analysis.** (**a**) Analysis of hnRNPs found in *Mbp* and *Mobp* mRNA granule. RT-qPCR analysis of hnRNPs in Aβ-treated and control cells (n=5). (**b, c**) hnRNPs western blot and relative quantification in total cell extracts from OLs (n≥3). (**d**) During granule transport, MBP mRNA is maintained in translationally silenced (inactive) or activated (active) states. (**e**) Representative binary micrographs of OLs showing the intracellular localisation of the different hnRNPs. Scale bar, 10 µm. (**f**) HnRNPA2-RIP workflow. Control IgG and anti-hnRNPA2 were used for RIP from oligodendrocyte lysates. (**g, h**) Analysis of RIP samples of *Mobp* and *Tau* mRNAs levels by RT-qPCR n=4). Data indicate means ± S.E.M and dots represent independent culture replicates. Statistical significance (*p<0.05, **p<0.01, ***p<0.001) was drawn by two-tailed paired Student´s t-test (**a, g, h**) and two-tailed ordinary one-way ANOVA followed by Dunnett’s post-hoc test (**c**).


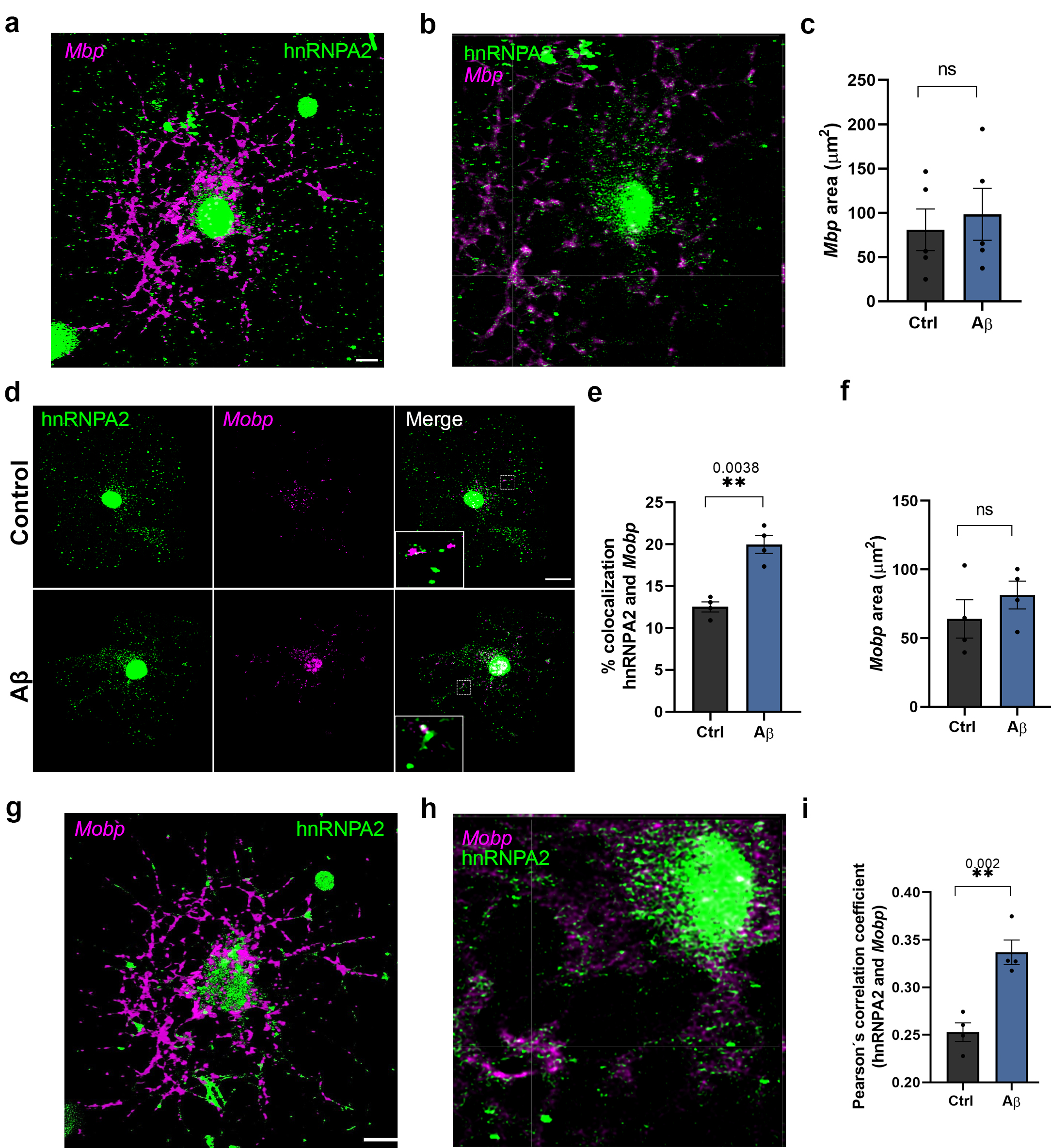


**Figure S5. RNAscope for *Mbp* and *Mobp* probes.** (**a**) Representative 3D image of hnRNP A2 (green) and *Mbp* (magenta)**.** Scale bar, 10 µm. (**b**) Representative orthogonal view of hnRNP A2 (green) and *Mbp* (magenta). (**c**) Total area occupied by *Mbp* transcripts in control and Aβ-treated OLs (n=4). (**d, e**) Representative confocal images showing hnRNP A2 (magenta) and *Mobp* (green) transcript and the colocalised image. *Mobp* transcripts were found in the cytoplasm and nucleus of OLs. Aβ-treated OLs show a higher percentage of colocalisation in the cytoplasm (n=4). Scale bar, 10 µm. (**f**) Total area occupied by *Mobp* transcripts in control and Aβ-treated OLs (n=4). (**g**) Representative 3D image of hnRNP A2 (green) and *Mobp* (magenta)**.** (**h**) Representative orthogonal view of hnRNP A2 (green) and *Mobp* (magenta). Scale bar, 10 µm. (**i**) Quantification of Pearson´s correlation coefficient for hnRNP A2 and *Mobp* (n=4). Data are represented as means ±S.E.M and dots indicate independent culture replicates. Statistical significance, *p<0.05, **p<0.01 was drawn by two-tailed paired Student´s t-test (**c, e, f, i**).


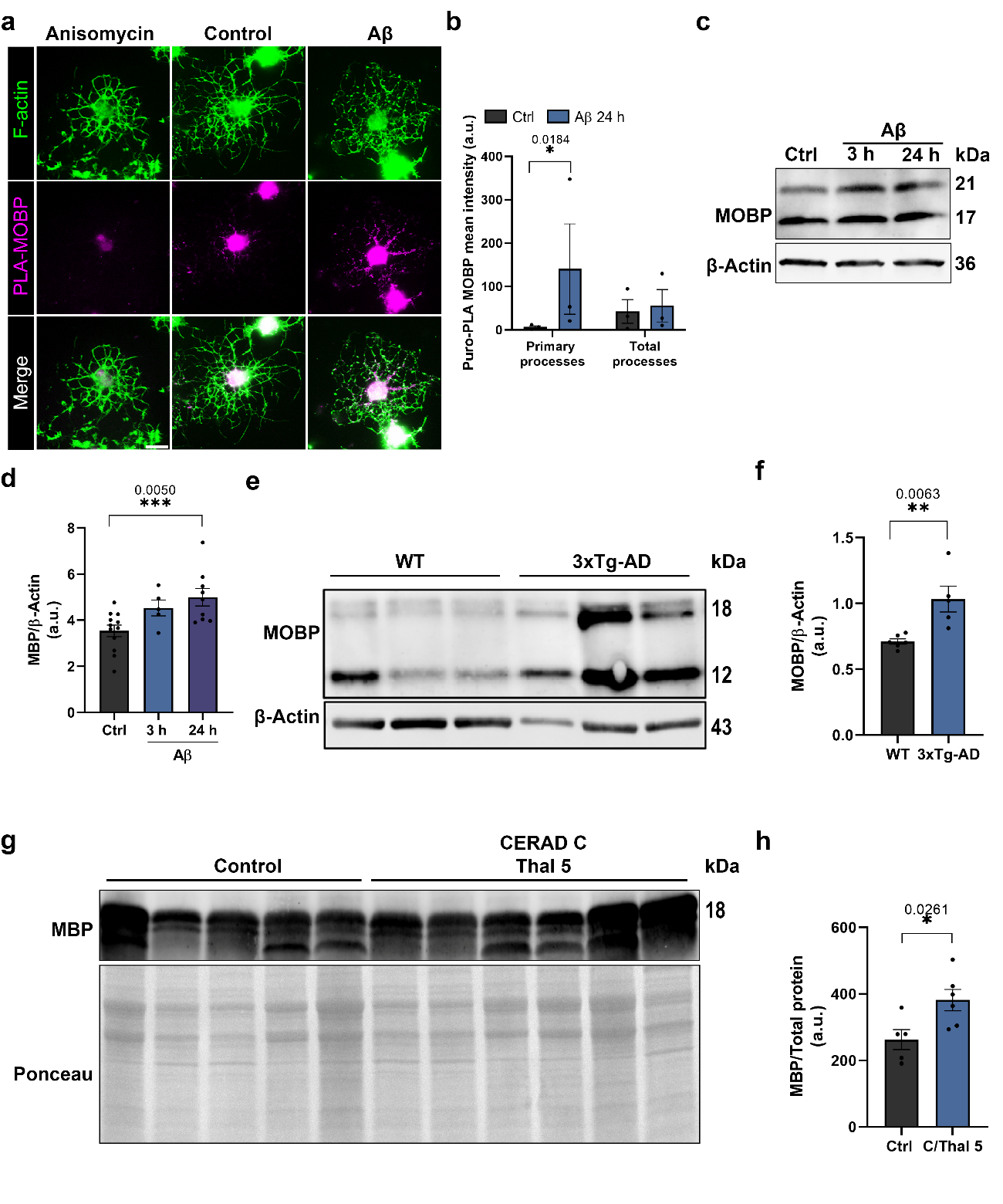


**Figure S6. Regulation of *Mobp* translation by Aβ.** (**a, b**) Photographs show MOBP puro-PLA-positive puncta in the soma, primary and total processes. MOBP PLA positive puncta was analysed in bins of 10 μm ranging from the soma in primary and total processes (n=3). Scale bar, 10 µm. (**c, d**) MOBP expression and relative quantification in oligodendrocyte cell extracts normalised to β-actin (n≥5). (**e, f**) MOBP expression and relative quantification in hippocampal lysates from 6-month-old WT (n=4) and 3xTg-AD (n=4) mice normalized to β-actin.(**g, h**) MBP expression and relative quantification in human hippocampal lysates from control (n=5) and AD patients (CERAD C and Thal 5) (n=6) normalized to total protein content (Ponceau S). Data are represented as means ±S.E.M and dots indicate independent culture replicates (**b, d**), individual animals (**f**) and individual patients (**h**). Statistical significance, *p<0.05, **p<0.01, ***p<0.001 was drawn by two-tailed paired (**b**), unpaired (**f, h**) Student´s t-test and one-way ANOVA followed by Dunnett’s post-hoc test (**d**).


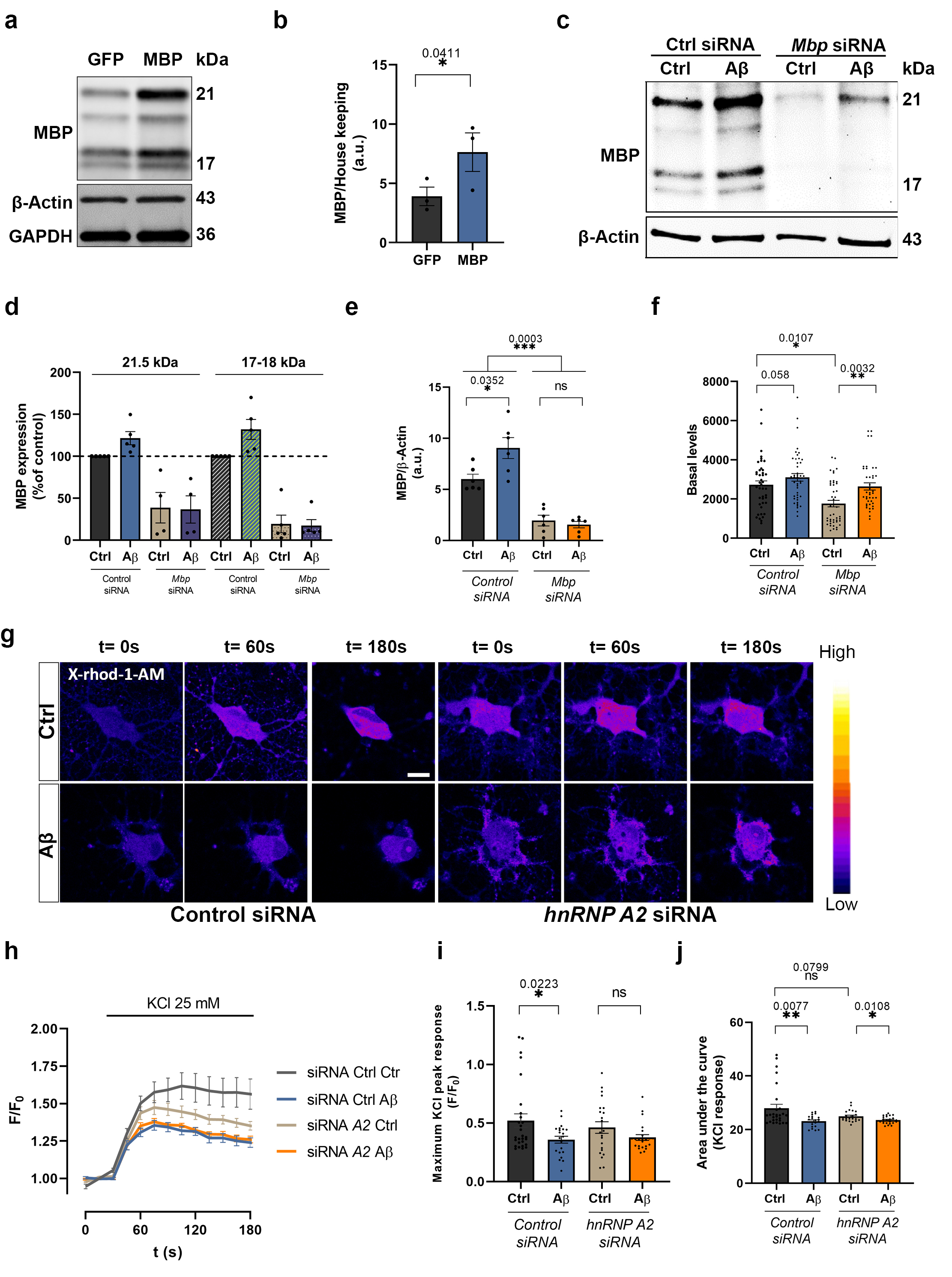


**Figure S7. Modulation of MBP expression and Ca^2+^ influx in hnRN A2-silenced OLs.** (**a, b**) Western blot and analysis of MBP expression levels in OLs infected with AAV8-pMBP-GFP and AAV8-pMBP-MBP-IRES-GFP (n=3). (**c, d, e**) Western blot and analysis of MBP expression levels following Aβ exposure in OLs treated with control siRNA and *Mbp*-targeting siRNA are shown (n≥4). (**f**) Basal levels are shown in the graph. (**g**) OLs transfected with either control siRNA or *Hnrnpa2b1*-targeting siRNAs were loaded with x-Rhod-1 AM and exposed to 1 µM Aβ for 24 h. Time course of intracellular Ca^2+^ levels were recorded before and after KCl 25 mM stimulus by confocal microscopy. Scale bar, 10 µm. (**h-j**) Graphs show the maximum peak and the area under the curve (AUC) of KCl response in the different conditions. Data indicate means ± S.E.M and dots represent independent culture replicates (**b, c, e**) or individual cells (**f, i, j**), *p<0.05, **p<0.01, ***p<0.001, compared to controls. Statistical significance was drawn by two-tailed paired (**b, d, e**) and unpaired (**f, i, j**) Student´s t-test.

**Supplementary table 1**

**Table S1.** Differential gene expression analysis of bulk RNA-seq from primary cultured oligodendrocytes treated with control or Aβ (1 µM, 24 h, n=3). The table shows the output of DESeq2 analysis, including gene names, log2 fold change, adjusted p-values (Padj), and base mean expression values. Genes with Padj < 0.05 were considered significantly differentially expressed.

**Supplementary table 2**

**Table S2.** Characteristic of controls and AD subjects categorised as stages I to VI of Braak and Braak, CERAD and Thal criteria. Western Blot: WB; Inmunofluorescence: IF.

| Ref. nº | Braak stage NFT | CERAD  Neuritic plaques | ThaL  Amyloid b  plaques | Gender | Age | Region analysed | Postmortem delay* | Analysis  #Cell |
| --- | --- | --- | --- | --- | --- | --- | --- | --- |
| 1423 | - | - | - | F | 82 | Hp | 5:00 | WB |
| 1378 | - | - | - | M | 78 | Hp | 6:00 | IF (#59 cells) |
| 1648 | - | - | - | M | 73 | Hp | 6:10 | IF (#16 cells) |
| 1536 | - | - | - | M | 79 | Hp | 4:45 | WB |
| 1733 | - | - | - | M | 76 | Hp | 11:30 | WB |
| 1423 | - | - | - | F | 82 | Hp | 5:00 | WB |
| 695 | I | - | - | M | 80 | Hp | 10:00 | IF (#38 cells) |
| 1697 | I-II | - | - | M | 78 | Hp | 6:00 | WB |
| 1687 | II | - | - | F | 69 | Hp | 12:00 | WB |
| 1405 | II | - | - | M | 80 | Hp | 5:30 | WB |
| 1431 | II | - | - | F | 97 | Hp | 20:00 | WB |
| 1247 | III | C | 5 | F | 80 | Hp | 8:00 | IF (#24 cells) |
| 1411 | III | C | 5 | F | 74 | Hp | 13:30 | WB |
| 1102 | IV | C | 5 | F | 84 | Hp | 3:25 | WB |
| 1286 | V | C | 5 | M | 79 | Hp | 5:00 | WB |
| 1585 | VI | C | 5 | F | 74 | Hp | 6:30 | WB |
| 1230 | VI | C | 5 | M | 79 | Hp | 4:15 | WB |
| 1456 | VI | C | 5 | F | 74 | Hp | 3:30 | WB |
| 1445 | VI | C | 5 | F | 73 | Hp | 3:30 | WB |
| 1645 | VI | C | 5 | F | 77 | Hp | 5:30 | WB |
| 977 | VI | C | 5 | M | 75 | Hp | 10:00 | IF (#39 cells) |
| 999 | VI | C | 5 | F | 76 | Hp | 10:00 | IF (#33 cells) |
| 1135 | VI | C | 5 | M | 79 | Hp | 6:25 | IF (#15 cells) |

**Supplementary table 3**

**Table S3.** Results of RIP-seq analysis to identify RNAs associated with hnRNP A2 in primary cultured oligodendrocytes treated with control or Aβ (1 µM, 24 h, n=3). The table is organized into two sections: (1) RNAs significantly enriched in the hnRNP A2 pulldown under control conditions, and (2) RNAs enriched after Aβ treatment. For each gene, log2 fold change, base mean expression, and adjusted p-value (Padj) are provided. Targets with Padj < 0.05 were considered significantly enriched.
